# Microscale sulfur cycling in the phototrophic pink berry consortia of the Sippewissett Salt Marsh

**DOI:** 10.1111/1462-2920.12388

**Published:** 2014-02-26

**Authors:** Elizabeth G Wilbanks, Ulrike Jaekel, Verena Salman, Parris T Humphrey, Jonathan A Eisen, Marc T Facciotti, Daniel H Buckley, Stephen H Zinder, Gregory K Druschel, David A Fike, Victoria J Orphan

**Affiliations:** 1 Department of Department of Microbiology Graduate Group, University of CaliforniaDavis, CA, 95616, USA; 2 Department of Evolution and Ecology, University of CaliforniaDavis, CA, 95616, USA; 3 Department of Microbiology and Immunology, University of CaliforniaDavis, CA, 95616, USA; 4 Department of Biomedical Engineering, University of CaliforniaDavis, CA, 95616, USA; 5 UC Davis Genome Center, University of CaliforniaDavis, CA, 95616, USA; 6 Arctic Technology, Shell Technology NorwayOslo, N-0277, Norway; 7 Department of Organismic and Evolutionary Biology, Harvard UniversityCambridge, MA, 02138, USA; 8 Department of Marine Sciences, University of North Carolina at Chapel HillChapel Hill, NC, 27599, USA; 9 Ecology and Evolutionary Biology, University of ArizonaTucson, AZ, 85721, USA; 10 Crop and Soil Sciences, Cornell UniversityIthaca, NY, 14853, USA; 11 Department of Microbiology, Cornell UniversityIthaca, NY, 14853, USA; 12 Department of Earth Sciences, Indiana University-Purdue UniversityIndianapolis, IN, 46202, USA; 13 Department of Earth and Planetary Sciences, Washington UniversitySt. Louis, MO, 63130, USA; 14 Division of Geological and Planetary Sciences, California Institute of TechnologyPasadena, CA, 91125, USA

## Abstract

Microbial metabolism is the engine that drives global biogeochemical cycles, yet many key transformations are carried out by microbial consortia over short spatiotemporal scales that elude detection by traditional analytical approaches. We investigate syntrophic sulfur cycling in the ‘pink berry’ consortia of the Sippewissett Salt Marsh through an integrative study at the microbial scale. The pink berries are macroscopic, photosynthetic microbial aggregates composed primarily of two closely associated species: sulfide-oxidizing purple sulfur bacteria (PB-PSB1) and sulfate-reducing bacteria (PB-SRB1). Using metagenomic sequencing and ^34^S-enriched sulfate stable isotope probing coupled with nanoSIMS, we demonstrate interspecies transfer of reduced sulfur metabolites from PB-SRB1 to PB-PSB1. The pink berries catalyse net sulfide oxidation and maintain internal sulfide concentrations of 0–500 μm. Sulfide within the berries, captured on silver wires and analysed using secondary ion mass spectrometer, increased in abundance towards the berry interior, while δ^34^S-_sulfide_ decreased from 6‰ to −31‰ from the exterior to interior of the berry. These values correspond to sulfate–sulfide isotopic fractionations (15–53‰) consistent with either sulfate reduction or a mixture of reductive and oxidative metabolisms. Together this combined metagenomic and high-resolution isotopic analysis demonstrates active sulfur cycling at the microscale within well-structured macroscopic consortia consisting of sulfide-oxidizing anoxygenic phototrophs and sulfate-reducing bacteria.

## Introduction

Microbial redox metabolism drives biogeochemical cycles and exerts a profound influence over the flux of energy throughout global ecosystems (Schlesinger, 1997; Falkowski *et al.*, 2008). Recent work in diverse environments, from the deep sea to the human gut, has demonstrated that many of these essential ecosystem processes are mediated not by a single species but by the metabolic interactions of syntrophic microbial consortia (Boetius *et al.*, 2000; Orphan *et al.*, 2002; Overmann and Schubert, 2002; Schink, 2002; Hansen *et al.*, 2011). Syntrophy, a mutualistic interaction based on the exchange of metabolites, allows microbes to exploit metabolic niches that are otherwise inaccessible to a single species (Overmann and van Gemerden, 2000; Schink, 2002; Orphan, 2009).

In closely associated microbial consortia, electron donors and acceptors are transferred over minute distances from cell to cell and drive micrometer-scale biogeochemical cycling. This tightly coupled metabolic activity occurs on spatiotemporal scales that often elude detection by traditional analytical approaches. However, these ‘cryptic’ transformations have major implications for the dynamics of biogeochemical cycling in the macroscale ecosystem (Canfield *et al.*, 2010; Holmkvist *et al.*, 2011; Stewart *et al.*, 2012). Understanding the structure and function of this intricate microbial metabolic network is essential for accurate modelling of biogeochemical cycles (Treseder *et al.*, 2011), prediction of the ecosystem dynamics following perturbations (Allison and Martiny, 2008), and interpretation of net contribution of microbial interactions to bulk measurements of the geochemical environment (Brüchert, 2004; Fike *et al.*, 2009).

**Fig 1:**
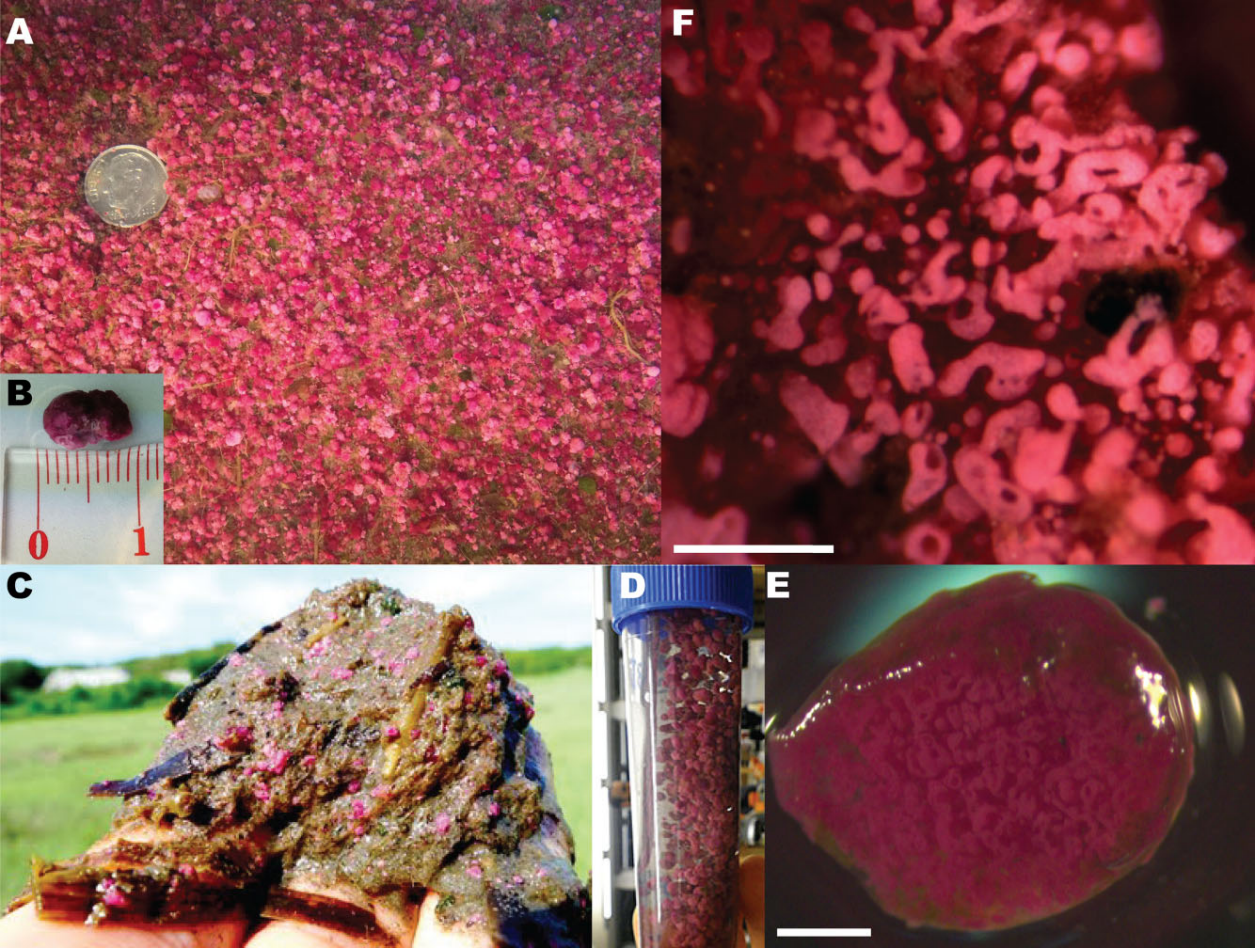
A. Intertidal pools in Little Sippewissett Salt Marsh form dense stands of pink berry aggregates at the sediment–water interface. B. Large aggregates can reach nearly a centimeter in size. C. Pink berries in sediment (0–5 cm) collected from an intertidal pool in Little Sippewissett. D. Berries can be easily washed free of marsh sediment and manipulated in the lab. E. Cross-section of a berry reveals pink tubules encased in a clear exopolymer matrix, scale bar is 0.5 mm. F. Higher magnification view of pink berry tubules, scale bar is 200 μm.

Photosynthetic microbial mats and aggregates provide an excellent system to investigate the influence of microbial metabolic interactions over biogeochemical processes at the micrometer scale (Canfield and Des Marais, 1993; Decker *et al.*, 2005; Baumgartner *et al*., 2006; 2009[Bibr b3],[Bibr b4]; Fike and Grotzinger, 2008; Fike *et al.*, 2008; Petroff *et al.*, 2011). The ‘pink berries’ (Fig. 1) are visually striking photosynthetic aggregates of uncultured microbes found in the Little and Great Sippewissett salt marshes (Falmouth, MA). Reaching up to a centimetre in diameter, these aggregates are found at the sediment–water interface of intertidal pools surrounded by tall-form smooth cordgrass (*Spartina alterniflora*). The berries have been studied for over three decades as a part of the Microbial Diversity summer course at the Marine Biological Laboratory in Woods Hole, MA (Gibson *et al.*, 1984). Initial characterizations revealed that the primary biomass of the aggregates is composed of anoxygenic phototrophs, purple sulfur bacteria of the family *Chromatiaceae* (Seitz *et al.*, 1993). Respiratory activity and the dense exopolymer matrix create anoxic conditions immediately below the aggregate surface, though net sulfide production had not been detected (Seitz *et al.*, 1993).

We show that the pink berries are formed by a consortium of purple sulfur bacteria (PSB) and sulfate-reducing bacteria (SRB) that form a specific interspecies association involving direct transfer of sulfur species. The present study of the pink berries utilizes a holistic approach to track sulfur metabolic interactions at the microbial scale, from draft genomes to ecophysiology. Investigation of community diversity and metabolic function using 16S rRNA gene surveys, microscopy and shotgun metagenomics generated hypotheses about the cycling of sulfur and exchange of nutrients within the aggregates. A suite of geomicrobiological tools from microvoltammetry to radio- and stable isotope approaches were employed to interrogate these predictions, illustrating the microbial dynamics and geochemical signatures of this cryptic microscale sulfur cycle.

## Results

### Phylogenetic diversity in the pink berry consortium: 16S rRNA gene surveys

**Fig 2:**
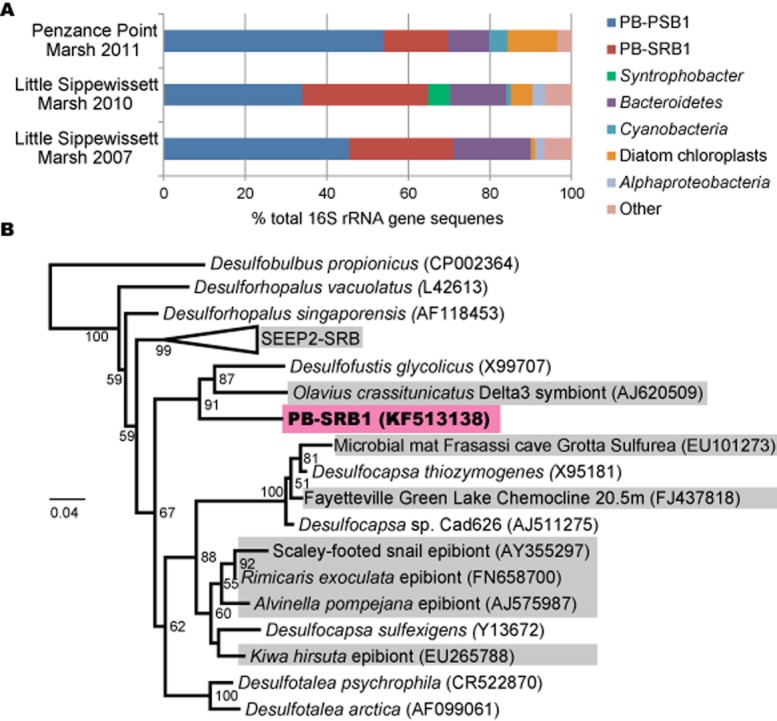
A. 16S rRNA gene sequences isolated from pink berries which were collected from Penzance Point marsh in 2011 (89 clones), and Little Sippewissett marsh in 2010 (94 clones) and 2007 (90 clones). Two operational taxonomic units (OTUs), a purple sulfur bacteria (PB-PSB1) and sulfate-reducing bacteria (PB-SRB1), account for more than 60% of the sequences observed. B. Maximum-likelihood phylogeny of the full-length PB-SRB1 OTU and related *Desulfobulbaceae*. Environmental sequences from uncultured organisms are shown in gray. Bootstrap support (500 replicates) greater than 50% are displayed at the nodes. Branch lengths (and scale bar) correspond to the mean number of nucleotide substitutions per site on the respective branch.

Analysis of 16S rRNA genes found in pink berry aggregates demonstrate a consistent, simple community where two phylotypes (defined at 97% sequence identity clustering) account for more than 65% of the sequenced clones (total of 273 sequences from three samples, Fig. 2A). PB-PSB1, the most abundant phylotype (35–53% of sequences), is an uncultured species of purple sulfur bacteria (PSB) belonging to the *Halochromatium–Thiohalocapsa* lineage of the *Chromatiaceae* (Supporting Information, Fig. S1).

The second most abundant phylotype, PB-SRB1 (15–35% of sequences), is most closely related to *Desulfofustis glycolicus*, a glycolate-oxidizing sulfate-reducer in the family *Desulfobulbaceae* (Fig. 2B). Uncultured organisms closely related to PB-SRB1 were often found in association with organisms that participate in sulfide-oxidizing symbioses or in environments dominated by sulfide oxidation (either chemotrophic or phototrophic, Fig. 2B). In addition to the dissimilatory reduction of sulfate, isolates from the genera *Desulfofustis* and *Desulfocapsa* have been shown to grow by the disproportionation of elemental sulfur and thiosulfate in the presence of an exogenous sulfide scavenger (Finster *et al.*, 1998; Finster, 2008).

The remaining 16S rRNA gene sequences are predominantly chloroplasts from marine diatoms and diverse phylotypes from the phylum *Bacteroidetes*. Dominant phylotypes of *Bacteroidetes* belong largely to clades of environmental sequences from areas of known or suspected active sulfur cycling, with few cultured representatives (Supporting Information, Fig. S2). Analysis of 18S rRNA gene sequences amplified from pink berries revealed several different phylotypes related to pennate diatoms and dinoflagellates (Supporting Information, Fig. S3). For the purposes of this work, we have focused further analysis on the dominant berry phylotypes, PB-PSB1 and PB-SRB1.

### In-situ identification and spatial arrangement

**Fig 3:**
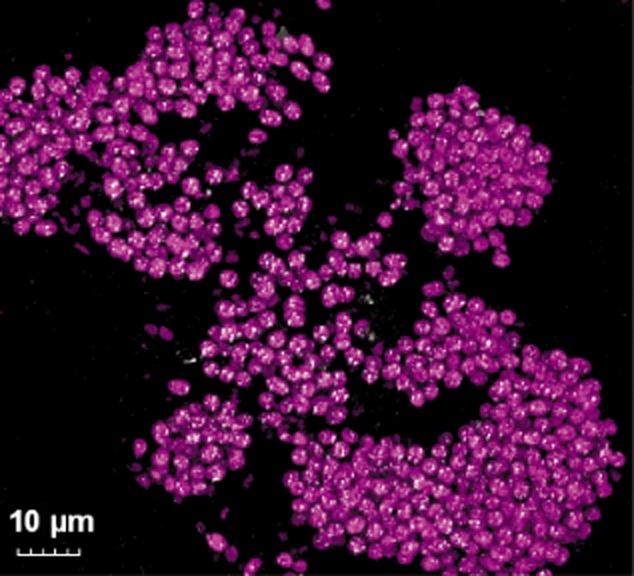
Epireflective confocal microscopy of sectioned pink berry tissue. Autofluorescence (excitation 543 nm, emission 550–570 nm) of the purple sulfur bacteria is shown in pink, and reflective signal from the refractile elemental sulfur inclusions is shown in white.

Pink berries are composed of irregularly shaped pink tubules in a transparent exopolymer matrix (Fig. 1F). Confocal microscopy of pink berry thin sections revealed that these tubules are dominated by purple sulfur bacteria, identified as autofluorescent cocci (2–4 μm in diameter) containing refractile elemental sulfur inclusions (Fig. 3). These refractile inclusions were labile in solvents (methanol, ethanol) and detergents (SDS, Triton X100), corroborating their identification as intracellular elemental sulfur globules. These cells were also fluorescently labelled by catalysed reporter deposition–fluorescence in situ hybridization (CARD-FISH) using the GAM42a group-specific probe which hybridizes to 16S rRNA from *Gammaproteobacteria* (Manz *et al.*, 1992; Supporting Information, Fig. S4).

**Fig 4:**
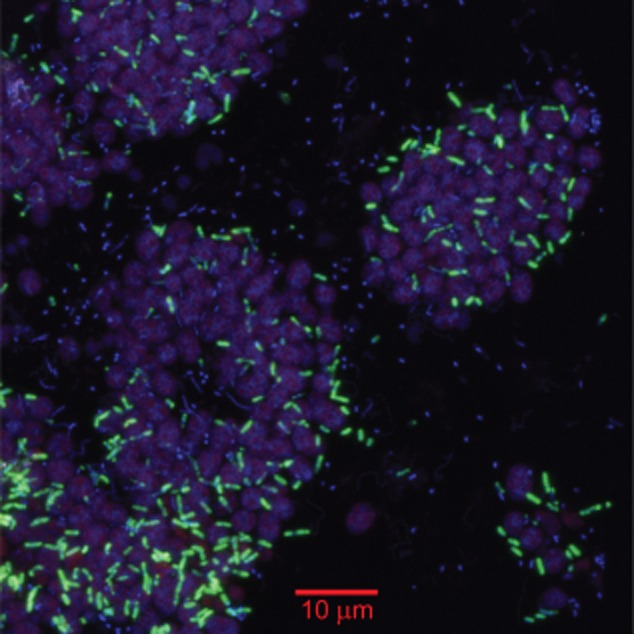
Identification of the berry-associated PB-SRB1 species by catalysed reporter deposition–fluorescence in situ hybridization (CARD-FISH) using a phylotype-specific probe. Scanning confocal micrograph shows a cross section of pink berry biomass from near the periphery of the aggregate. This image is an overlay of three fluorescent signals: autofluorescent purple sulfur bacteria (shown in pink, excitation 543 nm, emission 550–570 nm), CARD-FISH signal from the SRB-PiBe213 probe (shown in green, Alexa 488 tyramide) and DAPI nucleic acid stain (shown in blue).

PB-SRB1 species was localized via CARD-FISH with a phylotype-specific probe (SRB-PiBe213; this study) designed from the 16S rRNA gene libraries. The SRB-PiBe213 probe hybridized to 3-μm-long rods that were abundant and interspersed throughout the dense islands of PB-PSB1 cells (Fig. 4). A similar hybridization pattern was observed with a group-specific probe targeting the *Deltaproteobacteria* [DELTA495a-c; Lücker and colleagues (2007)]. Free cells hybridizing to the SRB-PiBe213 probe were rare (< 1% total cells) but could be detected in the overlying water of the intertidal pool (data not shown). Overlying pool water and the patches of pink sand in the nearby tidal channel were also found to contain microscopic aggregates of coccoid PSB cells in association with rod-shaped cells hybridizing to the SRB-PiBe213 probe. The smallest aggregations contained only 5–6 total cells, while larger microaggregates were 20–50 μm in diameter (data not shown).

### Metagenomic analysis of phylogenetic diversity

**Fig 5:**
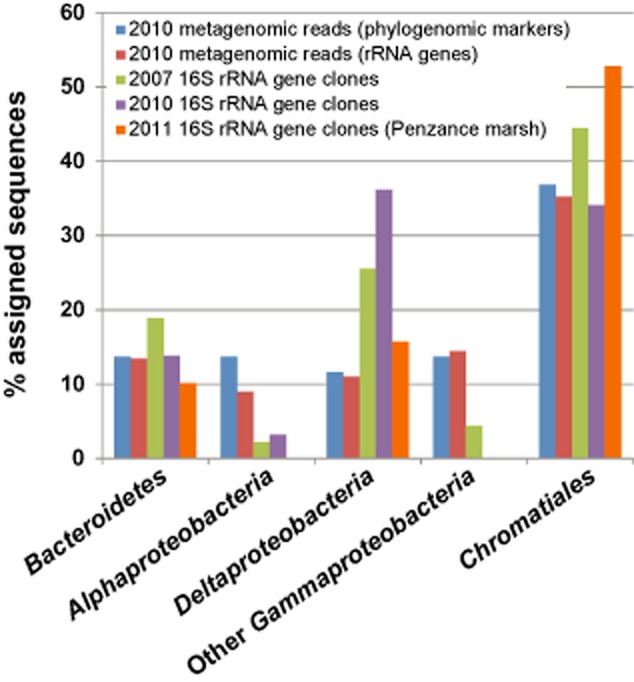
Comparison of bacterial diversity estimates from16S rDNA PCR-amplified clone libraries with unassembled 250 bp paired-end Illumina metagenomic reads. Metagenomic data were analysed using two different methods: maximum-likelihood read placement onto phylogenies conserved protein-coding marker genes (‘phylogenomic markers’) via Phylosift or the read's sequence similarity to ribosomal RNA genes via MG-RAST's M5RNA pipeline. A total of 42 351 metagenomic reads were classified as bacterial ribosomal RNA sequences, and 44 980 reads were assigned to bacterial phylogenetic marker genes.

The diversity of the microbes in the pink berries was also investigated using unassembled Illumina shotgun metagenomic data, which avoids the bias inherent in polymerase chain reaction (PCR) amplification. The abundance and diversity of different taxa in the metagenomic data were assessed using both ribosomal RNA gene sequences (Meyer *et al.*, 2008) and conserved phylogenetic marker genes (Darling *et al.*, 2014). The diversity observed in the shotgun metagenomic sequences recapitulated that of the PCR-based surveys (Fig. 5). A notable exception was the presence of significant numbers of *Alphaproteobacteria* in the shotgun data, driven by representatives of the *Rhodobacterales* (*Oceanicola* and *Oceanicaulis* species) and the *Rhodospirillales*. This discrepancy is likely due to bias introduced by the ‘universal’ 8F bacterial primer which poorly covers the organisms from these orders, according to in-silico specificity searches against the SILVA and Ribosomal Database Project (RDP) databases (data not shown).

Further metagenomic analysis of microbial diversity was conducted with co-assembled Roche 454 Titanium and Illumina HiSeq sequence data. Strategies optimized for community genomics (Peng *et al.*, 2012) were used to assemble 7400 scaffolds greater than 1 kilobase (kb; N50 1.6 kb, max contig 64 kb). Clustering of metagenomic scaffolds (see *Experimental procedures*) revealed two well-defined bins belonging to PB-PSB1 (20–25× coverage) and PB-SRB1 (15–20× coverage). The depth of coverage from these genomic bins corresponded to the abundance ratio of PB-SRB1 to PB-PSB1 observed in the 16S rRNA gene PCR data (1 PB-SRB1 RB per 1.1–1.7 PB-PSB1 cells). These genomic bins represent near complete genomes for both PB-SRB1 and PB-PSB1, as assessed by assembled sequence length, number of coding features and an analysis of 45 single copy phylogenetic marker genes (see Supporting Information, Table S1). In the present study, we have focused further analysis of these genomes on the pathways related to sulfur-based metabolism of these organisms.

### Metagenomic evidence for sulfur cycling

To assess the diversity of sulfur-cycling organisms in the berries, we mapped unassembled metagenomic sequence reads to the phylogeny of dissimilatory sulfite reductase genes (*dsrAB*, see *Experimental procedures*), which are widely used phylogenetic markers for both oxidative and reductive dissimilatory sulfur metabolisms (Loy *et al.*, 2013). Approximately 33% of reads aligning to the *dsrAB* sequence could not be taxonomically classified by this approach, likely because they originated from phylogenetically uninformative regions of the *dsrAB* genes. Most classified reads were placed in either the *Chromatiaceae* (44%) or *Desulfobulbaceae* (13%, Supporting Information, Fig. S5). Within these families, the bulk of the reads were assigned to *Halochromatium salexigens* (*Chromatiaceae*) and *Desulfofustis glycolicus* (*Desulfobulbaceae*), in congruence with the phylogenetic affiliation of the dominant 16S rRNA gene sequences. The remaining classified reads (10% of total aligned reads) were distributed amongst *Desulfobacteraceae* and *Desulfotomaculum*.

To further characterize the potential for sulfur redox cycling with the pink berries, we identified pathways for both sulfate reduction and sulfide oxidation from the complete assembled metagenomic dataset. While many of the same genes are used in both reductive and oxidative pathways, the homologues can be clearly distinguished both by divergent sequence and genomic context. All identified sulfur oxidative genes were found on scaffolds binned to PB-PSB1 by the independent sequence composition-based analysis, while sulfur reductive genes were located on scaffolds in the PB-SRB1 bin. Detailed discussion of the oxidative and reductive metabolic pathways in the PB-PSB1 and PB-SRB1 genomes is presented in the Supporting Information S1, Supporting Information S2, Table S2 and Figs S7–12

### Soluble sulfur geochemistry: speciation, abundance and isotopic composition

**Fig 6:**
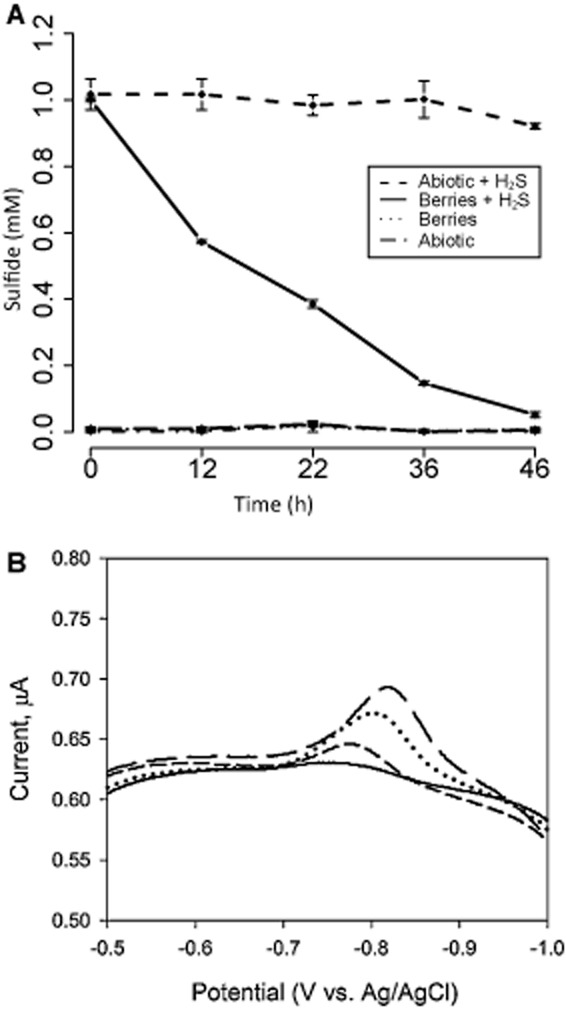
A. Sulfide concentrations measured by the Cline assay in microcosm incubations with 50 small berries in 50 ml of filter-sterilized marsh water. Incubations were kept on a 14 h light, 10 h dark cycle. Sulfide added to concentration of 1 mM was consumed over the course of the incubation in the presence of the berries (solid line), while the abiotic control showed no change (dashed line). Microcosms without added sulfide had no detectable change from an initial sulfide concentration near 0 mM. Error bars show the standard deviation of three biological replicate incubations. B. Voltammetric scans from a gold amalgam electrode inside a ∼ 0.5 cm diameter pink berry show a distinct peak relating to dissolved sulfide at ∼− 0.8 V (vs. Ag/AgCl) as the electrode tip penetrates the berry. As little control on the exact position of the electrode in the berry was possible, the data do not quantify a gradient of sulfide but rather outline a change in position and relative values across the aggregate. The solid line with no apparent peak was collected when the electrode was in the surrounding water and not penetrating the aggregate.

Illuminated microcosms of berries in anoxic filter-sterilized marsh water did not produce sulfide detectable by the Cline assay (Cline, 1969), but instead rapidly consumed 1 mM of added sulfide over the course of two days (Fig. 6A). To investigate the sulfur speciation and redox environment within the aggregates, we conducted cyclic voltammetry using a gold amalgam microelectrode inserted into a large berry (∼ 0.5 cm diameter). Voltammograms measured from the aggregate interior revealed a peak at a potential of 0.85 V (vs. Ag/AgCl), which changed as the electrode penetrated into and back out of the berry. This measurement corresponds to concentrations of sulfide between 13 and 41 μM at different points inside the berry (Fig. 6B; see also Supporting Information, Fig. S6). Independent analyses inside other large berries using a Clark-type sulfide microelectrode (Unisense, Aarhus, Denmark) indicated the presence of 5–20 μM of H_2_S (135–540 μM of total sulfide). No other electroactive species detectable by cyclic voltammetry (e.g. O_2_, Fe^3+^, Fe^2+^, Mn^2+^ and As^3+^, tetrathionate, dissolved/nanoparticulate elemental sulfur) were observed within the berries.

**Fig 7:**
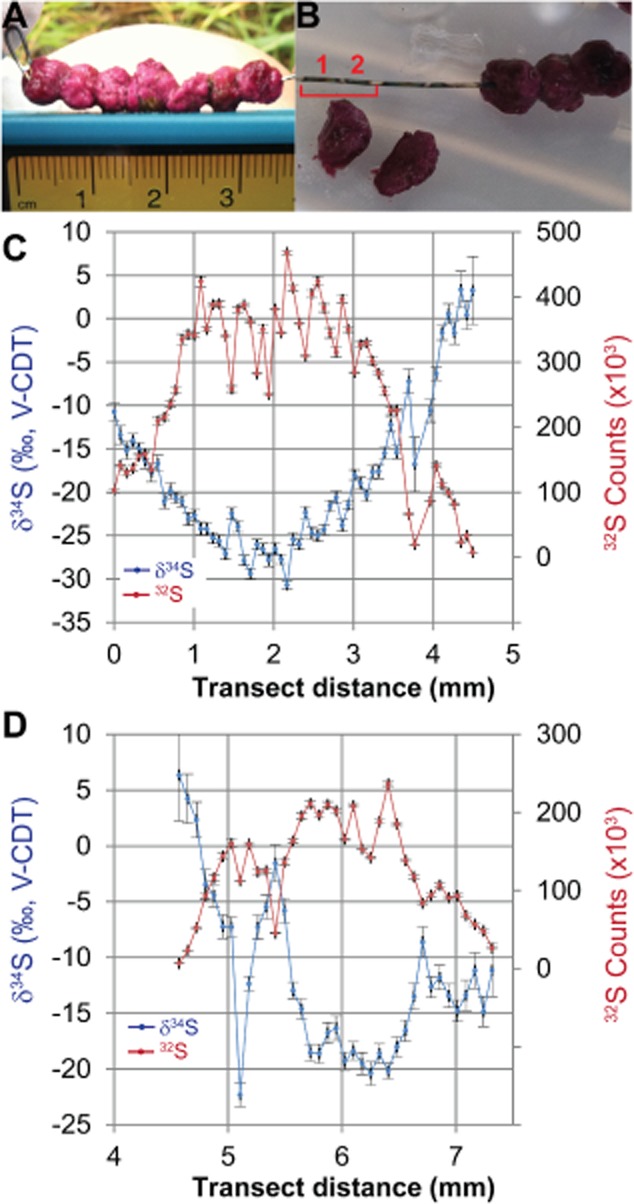
A. Large pink berries were threaded onto 24 gauge silver wire and incubated *in situ* overnight. B. Sulfide produced within the berry precipitated onto the wire surface, forming a thin film of AgS visible as the black metallic sheen where the berry had been. SIMS7f ion microprobe analyses were conducted in a transect (25 μm spot sizes) along the wire across the AgS films from two different berries marked by red numbers 1 and 2, shown in panels C and D respectively. C–D. Co-plotted on the y-axes are the sulfur abundance (^32^S counts, red) and δ^34^S (blue) for each point along the transect (x-axis). Vertical error bars represent the standard error (*n* = 20 cycles) for each measurement. The area between berries 1 and 2 where ^32^S counts approach 0 corresponds to the region on the wire where no dark sulfide film was visible. SIMS data for Fig. 7 have also been provided in the Supporting Information S2.

To investigate the isotopic composition of sulfide evolved within the berries, large aggregates (∼ 0.5 cm diameter) were threaded onto 24 gauge silver wires and incubated overnight *in situ* (Fig. 7A). Soluble sulfide was precipitated onto the wire as silver sulfide, leaving a dark tarnish with metallic sheen underneath each aggregate (Fig. 7B). Visible sulfide accumulation began just beneath the surface of the aggregate, with the darkest sulfide deposition towards the centre of the aggregate, confirming reports of sulfide from both microvoltammetry and sulfide microsensors. The abundance and isotopic composition of this precipitated silver sulfide was subsequently analysed using an IMS 7f-GEO magnetic sector secondary ion mass spectrometer (SIMS; CAMECA, Gennevilliers, France), following methods described by Fike and colleagues (2009). A transect along the wire that passed through two berries showed increasing ^32^S counts (sulfide abundance) and decreasing δ^34^S towards the centre of the aggregate (Fig. 7C). Where no visible sulfide deposition occurred (i.e. at berry edges or in between the berries), the ^32^S counts were also very low (Fig. 7C).

The δ^34^S of deposited sulfide ranged from ∼ 6‰ at the edge of the aggregates to −31‰ in the centre of one the aggregates. Sulfide deposited at the centre of the second berry in the transect showed lower ^32^S counts and less isotopic depletion (δ^34^S of −20‰) relative to the first berry (Fig. 7C). The water in the intertidal pond had a sulfate δ^34^S of +22‰; hence, these ‘intra-berry’ isotopic values indicate isotopic fractionations from ∼ 15‰ to 53‰. In contrast, silver wires that were incubated at the sediment surface nearby but not penetrating the berries (e.g. the loop at far left in Fig. 7A) had δ^34^S of −16 ± 2‰ (average ± SD, *n* = 15) uniformly distributed along the wire.

### Investigating sulfur cycling with stable isotope probing using ^34^SO_4_ and nanoSIMS

**Fig 8:**
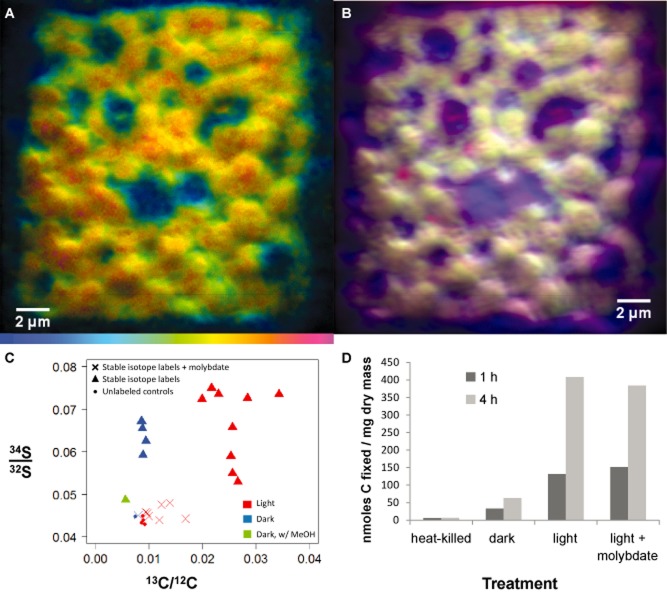
NanoSIMS analysis of pink berries isotopically labelled with ^34^S-enriched sulfate and ^13^C-enriched bicarbonate. A. Hue–saturation–intensity image mapping the ^34^S : ^32^S ratio. The color scale ranges from blue, set to the baseline ratio observed in unlabelled control conditions (0.044), to red, where the ratio is enriched ∼ 2 fold relative to baseline (0.08). Image shown is the composite eight consecutive 30 μm frames at 512 × 512 pixel resolution from a cross section of pink berry biomass from near the periphery of the aggregate. B. RGB composite image from overlaid primary ion signals showing the cellular arrangement of morphologically distinctive purple sulfur bacterial cells. Primary ion images overlaid are ^12^C ion (blue), ^12^C^14^N (green) and ^32^S (red). Underlying ion images for each mass available in Supporting Information S2. C. NanoSIMS-calculated mean incorporation of stable isotopically labelled ^13^C-bicarbonate (x-axis) and ^34^S-sulfate (y-axis) incubated in the dark (blue) or 12 h light/dark cycle (red) for 4 days under conditions of active sulfate-respiration (triangles) or SRB-inhibition with sodium molybdate (X), compared with unlabelled control incubations (dots). An isotope-labelled, dark-incubated berry was post-treated with methanol (green triangle). Values plotted are the averages of either 15 × 15 μm (light) or 30 × 30 μm (dark) rasters from different regions in total of seven aggregates (one per condition). D. ^14^C bicarbonate incorporation into acid-stable products in five berries incubated for either 1 h or 4 h with 1 mM sulfide. Incubations were conducted on heat-killed aggregates, dark equilibrated aggregates, and aggregates in the light with and without 10 mM molybdate additions.

To track the activity of the sulfur metabolism and cycling between members of the consortia, freshly collected pink berries were incubated anaerobically with ^34^S-enriched sulfate and ^13^C-enriched bicarbonate under both light and dark conditions. Enrichment of ^34^S in the morphologically distinctive PB-PSB1 cells was evident after 4 days of incubation (Fig. 8A and B). The ^34^S/^32^S ratio in these cells was approximately twice that observed in unlabelled control conditions. This signal was found to be labile in methanol (Fig. 8C), a solvent used for the quantitative extraction of elemental sulfur (Ferdelman *et al.*, 1997). Enrichment in ^34^S was documented in both light and dark incubations (Fig. 8C).

Accumulation of the ^34^S isotope label appears to be dependent upon the activity of the sulfate-reducing organisms in the aggregate; addition of sodium molybdate (NaMoO_4_), an inhibitor of sulfate reduction (Oremland and Capone, 1988), blocked the incorporation of the ^34^S-enriched label (Fig. 8C). Pronounced ^34^S-enrichment (1100 ± 200‰ for three replicate incubations) was observed in bulk berry biomass from incubations with ^34^S-enriched sulfate, as determined by elemental analyser–isotope ratio mass spectrometry (Supporting Information, Fig. S13). Unlabelled controls and molybdate-treated bulk biomass had isotopic composition comparable with that of native berries sampled directly from the marsh (δ^34^S values ranging from −15‰ to −24‰).

Carbon fixation was light-dependent, as assessed by both nanoSIMS measurements of ^13^C-incorporation (Fig. 8C) and radiolabelled (^14^C) bicarbonate incubation assays (Fig. 8D), indicating that PB-PSB1 acts as the primary producer in the aggregates. Molybdate incubations did not affect the light-dependent fixation of carbon in short-term incubations (1–4 h), indicating that PB-PSB1 is not directly inhibited by molybdate (Fig. 8D). In the 4-day incubations, molybdate was found to decrease the aggregates' carbon fixation in the light, suggesting that longer-duration molybdate treatment imposes sulfide limitation, thereby decreasing the primary productivity of PB-PSB1 (Fig. 8C).

## Discussion

Tightly coupled microbial consortia modulate biogeochemical cycles over spatiotemporal scales that often elude detection by traditional analytical approaches. The pink berries provide an accessible model system to investigate aggregate-associated ‘cryptic’ sulfur cycling, a process which likely plays a significant role in global biogeochemical transformations. In ocean systems, for example, abundant sulfide-oxidizing lineages (SUP05, SAR324, Agg47) have been reported in regions without detectable sulfide, from oxygen-minimum zones (Walsh *et al.*, 2009; Canfield *et al.*, 2010; Fuchsman *et al.*, 2011) to the oxygenated deep ocean (Swan *et al.*, 2011). Reports of these sulfide-oxidizing lineages in association with sinking organic particles (marine snow) suggests a syntrophic partnership with sulfate-reducing species, though such interspecies transfer of reduced sulfur compounds has not yet been demonstrated. Recent work has also suggested that interspecies sulfur transfer occurs in both consortia from acid mine drainage (Norlund *et al.*, 2009) and in syntrophic consortia responsible for the anaerobic oxidation of methane in the deep sea (Milucka *et al.*, 2012).

**Fig 9:**
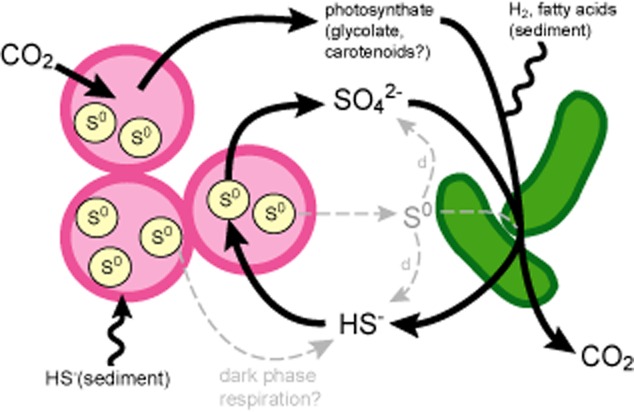
Model of sulfur cycling in the pink berry consortium. PB-SRB1 (green rods) reduce sulfate to sulfide, oxidizing a variety of electron donors from either exogenous sediment sources or from locally supplied photosynthate produced by PB-PSB1. PB-PSB1 (pink cocci) consume syntrophic sulfide, oxidizing sulfide to sulfate and intracellular stores of elemental sulfur (S^0^, pale yellow circles). Should PB-PSB1 cells lyse, intracellular sulfur might be reduced and/or disproportionated by PB-SRB1 (grey dashed arrows). Some electron donors for PB-PSB1 (HS^-^) and PB-SRB1 (H_2_ or fatty acids) are also likely provided exogenously by compounds effluxed from the sediment (squiggly lines). During the day, the phototrophic PB-PSB1 fixes CO_2_ into biomass, while at night it may derive maintenance energy by respiring elemental sulfur and intracellular carbohydrate reserves and producing sulfide. Though the PB-SRB1 genome suggests the genetic potential to fix CO_2_, results from our stable and radiocarbon experiments suggest PB-SRB1 does not contribute significantly to carbon fixation in the berries under the conditions of our incubations.

The pink berries, consortia made up primarily of a purple sulfur bacterial species (PB-PSB1, *Chromatiaceae*) and putative sulfate-reducing bacterial species (PB-SRB1, *Desulfobulbaceae*), provide a tractable system to begin decrypting microbial metabolic partnerships that drive sulfur cycling at the microscale. Based on the combination of phylogenetic information (i.e. the physiology of these species’ cultured relatives) and the conserved spatial structure of the pink berries, we hypothesized that intraberry sulfate reduction by PB-SRB1 provides a local source of sulfide for PB-PSB1 (Fig. 9). Such an association would allow PB-PSB1 to maintain photosynthetic activity even when exogenous sulfide is low or absent, such as during peak light conditions when the chemocline shifts down in the sediment by several centimetres relative to its nighttime levels (Revsbech *et al.*, 1983; Dillon *et al.*, 2009). Electron donors driving sulfate reduction could either be locally supplied from photosynthate or exogenously from the sediment (Fig. 9). Metagenomic sequencing confirmed the genetic potential for this intraberry sulfur cycle; the complete metabolic pathways for sulfate reduction and sulfide oxidation were present in the genomes of PB-SRB1 and PB-PSB1 respectively.

To test this metabolic model, high-resolution geochemical methods (SIMS, nanoSIMS, microvoltammetry) were used to track the cryptic sulfur cycle from sulfur metabolite pools to cell-specific sulfur assimilation and biomolecules. Sulfur-34 stable isotope labelling allowed us to follow the flow of sulfur within the aggregate, from the reduction of sulfate by PB-SRB1, to interspecies transfer and incorporation into PB-PSB1 cells. After incubation with ^34^S-enriched sulfate, we observed punctate, solvent-labile sulfur-34 enrichment in PB-PSB1 cells (Fig. 8) that is consistent with accumulation of labelled sulfur in intracellular sulfur globules (Fig. 3).

This sulfur-34 enrichment of PB-PSB1 is unlikely to occur via direct incorporation of the isotopically labelled sulfate. The cellular sulfur of *Chromatiaceae* species is dominated by periplasmic stores of elemental sulfur (34% of total cell weight), which is exclusively derived from the reduced sulfur pool (Dahl and Prange, 2006). Furthermore, many *Chromatiaceae*, including the closest cultured relatives to PB-PSB1, are unable to assimilate sulfate (Dahl, 2008; Kumar *et al.*, 2009). Even in those species that can assimilate sulfate, this process is thought to be repressed during anoxic photolithotrophic growth (Neumann *et al.*, 2000; Sander and Dahl, 2008). During photo-organotrophic growth, the model *Chromatiaceae* species *Allochromatium vinosum* assimilates sulfate via the *cysTWA* sulfate ABC transporter and the *cysDN* assimilatory ATP-sulfurylase. Homologues of these genes were not found in the PB-PSB1 genome, and searches against the complete assembled metagenomic data recovered only distant sequence matches from genes more closely related to those from organisms in the *Bacteroidetes*, *Alphaproteobacteria* or *Desulfobulbaceae*.

As direct ^34^S-sulfate incorporation by PB-PSB1 cells is unlikely, we propose that PB-SRB1 reduced the ^34^S-sulfate label to sulfide, which was then taken up and re-oxidized to elemental sulfur by PB-PSB1 (Fig. 9). Consistent with this interpretation, we found that pink berries rapidly oxidized sulfide (Fig. 6A) and that accumulation of the ^34^S-label in PB-PSB1 was dependent upon the activity of the sulfate-reducing bacteria (Fig. 8C). Sulfur-34 enrichment was not observed when sulfate reduction was inhibited by sodium molybdate. While the application of ‘specific’ inhibitors (such as molybdate) to mixed microbial populations can have unintended consequences on non-target organisms (Oremland and Capone, 1988), we have verified that PB-PSB1 were not directly inhibited by molybdate by demonstrating infrared light-dependent carbon fixation during short (1–4 h) incubations in the presence of exogenous sulfide (Fig. 8D). In longer incubations where exogenous sulfide became limiting (4 days), molybdate treatment diminished the light-dependent fixation of carbon (Fig. 8C). This decrease in primary productivity with SRB inhibition supports the hypothesis that syntrophically supplied sulfide buffers PB-PSB1 from episodic sulfide deprivation.

Having established the importance of sulfate reduction in the pink berry consortia, we sought to describe the pools of reduced sulfur within the pink berries. The close physical association of PB-SRB1 and PB-PSB1 (Fig. 4) suggests that re-oxidation of reduced sulfur species occurs over very short spatiotemporal scales. Sulfide could not be detected by a previous study with Clark-type sulfide microelectrodes (Seitz *et al.*, 1993), and we were only able to occasionally measure micromolar concentrations of sulfide inside very large aggregates using microvoltammetry and Clark-type sulfide microelectrodes (Fig. 6B). The ephemeral nature of the sulfide pool supports our predictions for rapid sulfide consumption in this closely coupled metabolic partnership. Under the conditions we describe, the pink berries were observed to be net sulfide consumers. However, this balance between consumption and production is likely influenced by many factors (e.g. intensity and duration of the photoperiod, temperature, electron donor supply), and further studies are necessary to better elucidate determinants of sulfide flux in the consortia.

The most likely source of this sulfide detected within the berries is the reduction of sulfate by PB-SRB1. However, sulfide could also be produced by several other metabolic processes (Fig. 9), including: (i) reduction of elemental sulfur by PB-SRB1, (ii) disproportionation of elemental sulfur or thiosulfate by PB-SRB1 or (iii) PB-PSB1's dark phase respiration of stored carbohydrates and concomitant reduction of periplasmic elemental sulfur reserves, producing polyhydroxyalkanoates and sulfide (van Gemerden, 1968; Rothermich *et al.*, 2000). To further understand the geochemistry of the pink berry sulfur cycle, we characterized both the spatial distribution and stable isotopic composition of this intraberry sulfide.

Differences in stable isotope ratios between oxidized and reduced pools of sulfur are widely used for modern and paleoecological reconstructions of the sulfur cycle (Canfield and Teske, 1996; Habicht *et al.*, 1998; Canfield, 2001; Hurtgen *et al.*, 2005; Fike *et al.*, 2006; Fike and Grotzinger, 2008; Sim *et al.*, 2011a). Sulfide oxidation mediated by anoxygenic phototrophs (e.g. PB-PSB1) is thought to have minimal impact on the fractionation of sulfur isotopes (Ivanov *et al.*, 1977; Fry *et al*., 1984; 1985[Bibr b35],[Bibr b36]; Zerkle *et al.*, 2009). However, both sulfate reduction and sulfur disproportionation can produce sulfide that is significantly depleted in heavy stable isotopes relative to the starting pool of sulfate (Canfield, 2001; Sim *et al.*, 2011a). Though use of sulfur isotopic fractionation to make inferences about sulfur cycling is widespread, our understanding of the microbial metabolic information encoded in these measurements is based largely on pure-culture studies of single species (Kaplan and Rittenberg, 1964; Kemp and Thode, 1968; Sim *et al.*, 2011b) or on in situ geochemical measurements where the microbial community was poorly characterized (Habicht and Canfield, 2001; Brüchert, 2004).

The millimetre-sized pink berry consortia provided a well-constrained multispecies ecosystem in which to examine micron scale spatial variation in sulfur isotopic composition (δ^34^S_sulfide_). The use of sulfide capture on silver wire, a technique originally used for radiotracer ^35^S measurements (Cohen, 1984; Visscher *et al.*, 2000; Dubilier *et al.*, 2001), coupled with SIMS δ^34^S measurements, elucidated gradients of increasing sulfide concentration and isotopic depletion (lower δ^34^S values) from the periphery to the centre of large pink berries (Fig. 7). These isotopic geochemical gradients observed in the berries are similar to δ^34^S_sulfide_ trends observed from SIMS transects on silver discs through the oxycline of the Guerrero Negro photosynthetic mats (Fike and Grotzinger, 2008; Fike *et al.*, 2008; 2009).

The range of sulfide–sulfate fractionations observed in the berries (15‰ at the periphery to 53‰ at the aggregate centre) is consistent with values reported from pure cultures of sulfate-reducing bacteria which can range from ∼ 0‰ to 66‰ (Sim *et al.*, 2011a). Large isotopic fractionations, such as those observed in the centre of the berries, can be produced by either (i) slow-growing sulfate-reducing bacteria cultured with refractory or growth-limiting concentrations of electron donors (Sim *et al*., 2011a,b[Bibr b80],[Bibr b81]; Leavitt *et al.*, 2013), or (ii) step-wise fashion from the coupled metabolic activities of reductive and oxidative processes (e.g. phototrophic sulfide oxidation by PB-PSB1 or intermediate oxidation state sulfur disproportionation by PB-SRB1; see Supporting Information S1 for more detailed discussion of mechanisms that could have produced the observed isotopic gradients).

Cultured relatives of PB-SRB1 in the genera *Desulfofustis* and *Desulfocapsa* are capable of both sulfate reduction and the disproportionation of elemental sulfur or thiosulfate (Finster, 2008), corroborating our isotopic findings which suggest disproportionation could contribute to the pink berry sulfide pool. Both ecophysiological and genomic data strongly supports the conclusion that PB-SRB1 is capable of sulfate reduction, though genomic evidence for the disproportionation of elemental sulfur or thiosulfate by PB-SRB1 remains less clear. The exact suite of genes required for disproportionation is still unknown, despite the recent sequencing of *Desulfocapsa sulfexigens,* a sulfur disproportionator incapable of sulfate reduction (Finster *et al.*, 2013). The sulfur disproportionation pathway appears to involve many of the same genes used in sulfate reduction, in addition to a sulfite-oxidoreductase enzyme (Frederiksen and Finster, 2003) for which there are several candidates in genomes of PB-SRB1 and *D. sulfexigens* (Finster *et al.*, 2013).

In pure culture, growth by elemental sulfur disproportionation has been described only in the presence of a sulfide sink that keeps the exogenous sulfide concentration low, providing thermodynamically favourable conditions for this reaction (Canfield *et al.*, 1998; Finster, 2008). Phototrophic consumption of sulfide by PB-PSB1 provides a biotic sulfide sink in the consortia that could create an attractive niche for elemental sulfur disproportionation, with both abundant sulfur deposits and low concentrations of sulfide. Should sulfide reach higher concentrations, disproportionation of polysulfides could become an important portion of the metabolic strategy of PB-SRB1. Recent studies of anaerobic methane-oxidizing consortia suggest that at least some sulfate-reducing consortia members can disproportionate disulfide that forms in sulfidic environments by the abiotic reaction of elemental sulfur with sulfide (Milucka *et al.*, 2012).

## Conclusion

Tracking the interspecies metabolic exchanges that drive microscale biogeochemical processes in natural microbial communities remains a technical challenge. For example, biogeochemical cycling in laminated photosynthetic microbial mats have been studied for decades (e.g. Canfield and Des Marais, 1993; Decker *et al.*, 2005; Baumgartner *et al.*, 2009; Petroff *et al.*, 2011), yet the microbial interactions controlling substrate flux (Burow *et al.*, 2013) and geochemical signatures (Fike and Grotzinger, 2008; Fike *et al.*, 2008) are only just beginning to be understood. Using the pink berries, we demonstrate how an integrative microbiological and microgeochemical approach can be used to decrypt the microbial metabolic partnerships that drive sulfur cycling at the microscale. This methodology, which may ultimately be used to examine more complex ecosystems, offers direct evidence of syntrophic interspecies sulfur transfer.

SIMS analysis of spatial gradients in sulfide abundance and isotopic composition provide a geochemical context for this microscale sulfur cycle. We find it notable that, even within this well-described, limited diversity system, the observed isotopic variation in sulfide can be explained by a number of different plausible scenarios from sulfate reduction alone to a series of coupled reductive and oxidative processes (see also Supporting Information S1). This ambiguity indicates that care should be taken when using such data alone for reconstructions sulfur cycling in more complex sedimentary systems, both ancient and modern. While we have demonstrated the activity of one portion of the sulfur cycle (sulfate reduction and sulfide oxidation), data from our work and other sulfur-based syntrophies (Norlund *et al.*, 2009; Milucka *et al.*, 2012) suggest there may also exist a network of metabolic interactions involving intermediate oxidation state sulfur species (e.g. elemental sulfur, disulfide, thiosulfate) that remain to be explored.

## Experimental procedures

### Sampling

Unless otherwise stated, berries were sampled in June and July of 2007, 2010 and 2011 from a single intertidal pool formed in the Little Sippewissett Salt Marsh, Falmouth, MA USA (41°34′33.01′N, 70°38′21.24′W). Large berries (∼ 0.5–1 cm in diameter) used for the silver wire sulfide capture and cyclic microvoltammetry were sampled from a second pool in Little Sippewissett (41°34′33.52′N, 70°38′9.71′W) in September 2012. Pink berries were also sampled from Penzance Point Marsh, Woods Hole, MA, USA (41°31′29.95′N, 70°41′7.48′W) and were sampled in the summer of 2011. Berries were collected from the sediment–water interface by sieving (1 mm mesh size) and were washed three times in 0.2 μM of filter-sterilized marsh water.

### SSU rRNA clone libraries and sanger capillary sequencing

Five to 10 small aggregates were homogenized with PCR-grade water, and DNA was extracted with the MoBio PowerSoil kit (MoBio, Carlsbad, CA, USA) with a 1 min bead-beating step employed for lysis in place of the 10 min vortexing step outlined in the manufacturer's protocol. Bacterial 16S rRNA genes and eukaryotic 18S rRNA genes were PCR-amplified, cloned, sequenced and clustered to operational taxonomic units (OTUs) at the 97% sequence similarity threshold as described in the Supporting Information S1. GenBank accession numbers for dereplicated, chimera-checked 16S rRNA gene sequence data are KF512914–KF513148, and 18S rRNA gene sequence data are KF516997–KF517018. Phylogenic trees were constructed either with RAxML 7.2.8 (Stamatakis, 2006) with 1000 rapid bootstrap inferences and GTRGAMMA rate approximation, or FastTree using the approximate maximum-likelihood method (Price *et al.*, 2010) with 1000 SH-like support, GTRCAT approximation with 20 rate categories.

### Metagenomic sequencing and analysis

#### Metagenomic sequencing

Total extracted community DNA was sequenced by Roche GS 454 FLX+, Illumina HiSeq and Illumina MiSeq technologies as described in Supporting Information S1. All quality-filtered, unassembled sequence data is available under NCBI BioProject PRJNA214436 in the Sequence Read Archive (SRA) accessions SRX332170, SRX332174 and SRX332175. Data are also available via the MG-RAST server under MG-RAST IDs 4454153.3, 4517592.3 and 4516362.3. Overlapping Illumina MiSeq paired-end reads (250 bp) were assembled, when possible, using the MG-RAST 3.3 pipeline and classified using the M5RNA database to provide a diversity description paralleling that of the 16S rRNA gene clone libraries (Meyer *et al.*, 2008). To assess diversity of the *dsrAB* sulfur cycling marker genes, a Hidden Markov Model (HMM) and full-length reference tree were built from a curated *dsrAB* alignment (Loy *et al.*, 2009) using the HMMER 3.0 and FastTree (Price *et al.*, 2010) as implemented by the Phylosift pipeline (Darling *et al.*, 2014). Using the Phylosift pipeline, Illumina sequence reads were scanned using LAST (Kielbasa *et al.*, 2011), aligned to the reference marker with HMMER 3.0, and placed onto the full-length *dsrAB* phylogenetic tree using pplacer (Matsen *et al.*, 2010).

#### Metagenomic assembly, genomic binning and genome completeness

Roche 454 Titanium and interleaved paired-end Illumina HiSeq data were co-assembled using the idba_ud algorithm 1.0.9 (Peng *et al.*, 2012). Using protein queries from *Desulfobulbus propionicus* and *Allochromatium vinosum* tBLASTn searches identifying key sulfur-cycling functional genes were conducted on nucleotide databases of this complete, assembled metagenomic data. This dataset have been deposited as a whole genome shotgun (WGS) project at DDBJ/EMBL/GenBank under the accession AVFP00000000. The version described in this paper is version AVFP01000000. Data is also available via the MG-RAST database under MG-RAST ID 4532235.3.

Contigs larger than 1 kb were binned into genomes by tetranucleotide frequencies using emergent self-organized maps as described in Supporting Information S1 and previously (Dick *et al.*, 2009; Wrighton *et al.*, 2012). RAST annotations of the PB-PSB1 and PB-SRB1 genomes are available at FigShare (http://dx.doi.org/10.6084/m9.figshare.770903). This draft genome project has been deposited as a WGS project at DDBJ/EMBL/GenBank under the accessions AVFQ00000000 and AVFR00000000. The versions described in this paper are version AVFQ01000000 and AVFR01000000.

### Embedding and cryosectioning for microscopy and nanoSIMS

Washed berries were rinsed in phosphate-buffered saline (PBS) for 1 min and then fixed for 1 h at room temperature (4% paraformaldehyde, 0.5% glutaraldehyde in PBS). Post-fixation, berries were washed three times with PBS and incubated for > 1 h in a cryoprotectant solution (7.5% sucrose in PBS). Berries were then transferred to O.C.T. TissueTek (Sakura, CA, USA) and allowed to infiltrate for > 2 h before being flash frozen in liquid nitrogen. Frozen tissue blocks were sectioned at 10 μm thickness at −20°C using a straight-razor cryostat. Samples for CARD-FISH hybridization and epireflective confocal microscopy were placed onto Tissue Path Superfrost Plus Gold Slides (Fisher Scientific, Waltham, MA, USA), while samples for nanoSIMS were placed onto either glass rounds or indium tin oxide (ITO)-coated glass squares (Dekas and Orphan, 2011).

### CARD-FISH, imaging elemental sulfur inclusions and confocal microscopy

The probe design tool of the ARB software package (Ludwig *et al.*, 2004) was used to create specific probes for PB-SRB1 phylotype found in the 16S rRNA gene clone library data. Specificity of the probe (SRB-PiBe213) for this sequence cluster was checked against the GenBank database using BLAST, the SILVA database (release 108), the Ribosomal Database Project and NCBI database, and no hits for sequences outside of the target cluster could be detected. The SRB-PiBe213 probe (5′-tcctcctcgcacaaccgc-3′) was ordered as conjugate from Biomers (Ulm, Germany). The general gammaproteobacterial probe GAM42a probe sequence was checked and found to target the PB-PSB1 sequences.

In addition to hybridizations with the custom SRB-PiBe213 probe, GAM42A (Manz *et al.*, 1992) and Delta495a-c with competitors a-c (Lücker *et al*., 2007), the eubacterial probe EUB338I-III (Amann *et al.*, 1990; Daims *et al.*, 1999) was used as a positive control and a nonsense probe NON338 (Wallner *et al.*, 1993) as a control for nonspecific binding. Hybridizations and tyramide signal amplification were performed as described previously (Ishii *et al.*, 2004) with the modifications described in detail in the Supporting Information S1. Initial imaging was conducted using a Zeiss Axio IMAGER MZ epifluorescence microscope equipped with a color camera (AxioCam HRc, Carl Zeiss, Thornwood, NY, USA). Confocal microscopy of the SRB-PiBE213 CARD-FISH hybridization was conducted using a Leica TCS SP8X confocal microscope (Leica Microsystems, Wetzlar, Germany) with a tunable white light laser.

Elemental sulfur inclusions in the purple sulfur bacterial cells were imaged by epireflective confocal microscopy using an Olympus FV1000 LSM on tissue sections as described previously (Pasteris *et al.*, 2001). Briefly, the 488 nm laser line was used to generate the reflection signal from the refractile elemental sulfur granules with a detection window of 478–498 nm, while the autofluorescence from the purple sulfur bacterial cells was collected using the 543 nm laser line and automatic filter settings for Alexa546 fluorophore.

### Sulfide consumption assay

Triplicate microcosms were established with 50 small berries each (∼ 1–3 mm diameter) in 50 ml serum bottles of anoxic filter-sterilized in-situ marsh water under an N_2_/CO_2_ headspace (90:10), with or without sulfide added to a final concentration of 1 mM. Triplicate abiotic controls with and without 1 mM sulfide were also established and contained only anoxic filter-sterilized *in situ* marsh water. Microcosms were incubated on a 14 h light/10 h dark cycle at 28°C, and soluble sulfide was monitored spectrophotometrically (Cline, 1969).

### Cyclic microvoltammetry

Voltammetic analysis of berries collected in a 50 ml Falcon tube were performed utilizing glass Au-Hg amalgam electrodes (with tips drawn to ∼ 500 μm diameter) lowered vertically in 0.1–1 mm increments using a manual micromanipulator. The electrodes were constructed and calibrated according to the methods outlined by Brendel and Luther (1995). Electrodes were calibrated in the lab for O_2_, HS^-^ and Mn^2+^ using water collected from the sample site; each electrode's response was checked immediately before use by measuring the signal for 200 μM Mn^2+^ and calibrated using the pilot ion method after Meites (1965) and Slowey and Marvin-DiPasquale (2012). The tip of the electrode was positioned to penetrate a number of berries using this technique. The berries typically compressed slightly before the electrode tip was able to penetrate, followed by movement of the berry itself with the electrode as it penetrated. As such, exact spatial reference was not possible, although it was clear that a number of scans did occur inside individual berries. A sequence of 10 cyclic voltammograms was obtained at each position using a DLK-60 potentiostat and software (Analytical Instrument Systems, Flemington, NJ, USA). Instrumental variability between measurements was typically < 1%. Sulfide was also independently measured in other aggregates using a Unisense sulfide microsensor (Unisense, Aarhus, Denmark) according to manufacturer's instructions, with total sulfide calculated assuming an interior pH of 8 (Seitz *et al.*, 1993).

### Sulfide capture and SIMS 7f-Geo ion microprobe analysis of δ^34^S

Large berries (0.5–1 cm diameter) were collected, rinsed free of sediment, threaded onto 24 gauge silver wire (99.95%; Surepure Chemetals, Florham Park, NJ, USA) and incubated *in situ* from 4 p.m. until 11 a.m. the next day. Berries were then removed, wires were rinsed with de-ionized water and stored under a nitrogen atmosphere prior to analysis. The wire was sectioned, mounted onto a glass round using double-sided carbon tape and sputter-coated with 10 nm of gold. Analysis of silver sulfide precipitated on metallic silver was conducted using the Cameca IMS 7f-GEO magnetic sector SIMS at the Caltech Center for Microanalysis using methods described previously (e.g. Fike and Grotzinger, 2008; Fike *et al.*, 2008; Fike *et al.*, 2009) and in detail in the Supporting Information S1.

### Stable isotope amendment experiments

Anoxic, filter-sterilized in-situ marsh water was amended with ^13^C-enriched bicarbonate (98 atom % ^13^C, Isotec Sigma-Aldrich, St. Louis, MO, USA) at a 10 mM final concentration, and ^34^S-enriched sulfate (90 atom % ^34^S, Isotec Sigma-Aldrich) at a 28 mM final concentration, on top of the concentrations naturally present in the water (sulfate initially measured at 26.5 mM). Berries were washed free of sediment and placed into serum bottle incubations with sulfide added to 0.5 mM and an N_2_/CO_2_ (90:10) headspace. Incubations were maintained for 4 days at 28°C either in the dark, or the light (14 h light/ 10 h dark cycle). Similar light/dark incubations were also established in the presence of 10 mM of sodium molybdate to inhibit the activity of sulfate-reducing bacteria in the aggregates. Isotopically unlabelled light and dark control incubations were incubated in parallel and consisted of equivalent additions of standard isotopic composition bicarbonate and sulfate on top of that naturally present in the marsh water.

After 4 days, berries were removed from the incubations, fixed, embedded and sectioned for nanoSIMS analysis. Berries from the light condition were sectioned onto glass rounds and subsequently sputter-coated with 10 nm of gold. Continuous dark incubations were sectioned onto conductive ITO squares (Dekas and Orphan, 2011). Several berries from three dark biological replicates (+/− molybdate, and unlabelled controls) were fixed and flash frozen without embedding for analysis by bulk elemental analyser isotope ratio mass spectrometry (EA-IRMS).

#### NanoSIMS sample preparation and imaging

Samples for nanoSIMS analysis were optically mapped using phase contrast and epifluorescence microscopy to identify islands of PB-PSB1 target cells. Neither FISH nor CARD-FISH were conducted on these samples to preserve the intracellular elemental sulfur inclusions, which were found to wash out during permeabilization steps required for these protocols. Target PB-PSB1 cells were found to have abundant intracellular sulfur inclusions prior to nanoSIMS analysis.

Measurements were conducted on the Cameca NanoSIMS 50 L instrument at the Caltech Center for Microanalysis in two separate 3-day sessions in 2010 and 2011. Samples were collected using a primary Cs+ ion beam at 1.2 pA corresponding to a nominal spot size of ∼ 50 nm. The beam was rastered at either 256 × 256 or 512 × 512 pixel resolution over square regions from 15 to 30 μm in size. All samples were presputtered for 10–15 min to locally remove any exterior layers or gold coating. Seven secondary ions were simultaneously collected, ^12^C^-^, ^13^C^-^, ^12^C^14^N^-^, ^12^C^15^N^-^,^31^P^-^, ^32^S^-^, ^34^S^-^. NanoSIMS images were processed using ‘Open MIMS’, a plug-in to ImageJ available online at http://www.nrims.hms.harvard.edu/NRIMS_ImageJ.php (Gormanns *et al.*, 2012). Each series of frames was corrected for drift and detector dead time but represent raw values that were not corrected for instrumental mass fractionation. Values shown are the sum of several frames from each analysis region.

### Bulk analysis of sulfur isotopes by EA-IRMS

Sulfate from in-situ marsh water was precipitated with barium chloride (Kolmert *et al.*, 2000), dried and prepared for EA-IRMS. Pink berries preserved from triplicate incubations (dark, +/− isotope labels, +/− 10 mM sodium molybdate) were dried at 55°C for 48 h, and crimped in sealed aluminum foil. Samples were analysed for their sulfur isotopic composition using an ECS 4010 elemental analyser (Costech Analytical Technologies, Valencia, CA, USA) coupled to a Thermo Finnigan Delta V Plus mass spectrometer (Thermo Scientific, Waltham, MA, USA). Sulfur isotope composition was calibrated against NBS-127, IAEA-S1, and IAEA-S3. Sulfur isotope values are reported in per mil (‰) relative to the V-CDT (Vienna Canyon Diablo Troilite) scale. Based on replicate analyses across several days, reproducibility of these sulfur isotope measurements is < 0.3‰ (1σ).

### Radiocarbon assay of carbon fixation

Incubations of five sediment-free 2 mm diameter berries were placed in small screw-cap tubes with 1 ml of filter-sterilized in-situ marsh water and an N_2_/CO_2_ headspace (90:10). Duplicate incubations were pre-equilibrated for 3 h either in the ambient light, dark or with 10 mM of final concentration sodium molybdate. Duplicate vials were also prepared with heat-killed berries (10 min boil). Just before the start of the experiment, each incubation was amended with neutralized sulfide to a final concentration of 1 mM, followed by the addition of 10 μL of ^14^C bicarbonate in 0.01 M of sodium hydroxide with a nominal specific activity of 250 μCi/ml. Incubations were sampled after either 1 or 4 h at room temperature illuminated by an array of 196 infrared light emitting diodes (λ = 850 nm) to ensure that any observed carbon fixation came from the purple sulfur bacteria and not from oxygenic phototrophs (diatoms, cyanobacteria) in the berries.

Incubations were terminated by the addition of 1 ml of saturated urea solution and heating to 85°C for 30 min to inactivate and disaggregate the berries. Unincorporated radioactive bicarbonate was removed by heating 50 μL of this berry homogenate with 400 μL of glacial acetic acid in glass scintillation vials at 65°C for 20 min. Vials were gently tapped throughout the heating to ensure removal of any condensate near the rim. Ten millilitres of Universol scintillation cocktail was added to the sample and the acid-stable products in the biomass were quantified as scintillation per second with an LS 650 multipurpose scintillation counter (Beckman Coulter, Fullerton, CA, USA).

## Supporting Information

S1 Supplemental results and discussion. **S2.**  Supplemental data. SIMS 7f-GEO raw and processed data used to construct for main text Figure 7C,D. **S3.**  Supplemental data. NanoSIMS-50L raw and processed data used to construct Figure 8A,B in main text.

Fig S1 Maximum-likelihood phylogeny (rooted) constructed from 16S rRNA gene sequences of pink berry purple sulfur bacteria PB-PSB1 and cultured *Chromatiales* relatives. Phylogeny was constructed with FastTree using the approximate maximum-likelihood method (Price *et al*., 2010) and GTRCAT approximation with 20 rate categories. Values at the nodes are SH-like local supports (1000) computed by FastTree and used as condiference values of tree branches. Scale bar represents the number of estimated changes per position for a unit of branch length. **Fig. S2.** Maximum-likelihood phylogeny (mid-point rooted) constructed from 16S rRNA gene sequences of pink berry *Bacteroidetes* phylotypes OTUs (pink), cultured isolates (blue) and uncultured environmental amplicon. Phylogeny was constructed with FastTree using the approximate maximum-likelihood method (Price *et al*., 2010) and GTRCAT approximation with 20 rate categories. Values at the nodes are SH-like local supports (1000) computed by FastTree and used as condiference values of tree branches. Scale bar represents the number of estimated changes per position for a unit of branch length. Marked with red stars are the dominant pink berry phylotypes. Note the relative scarcity of cultured isolates and abundance of related sequences from diverse sulfur-cycling habitats. **Fig. S3.** Maximum-likelihood phylogeny (mid-point rooted) constructed from 18S rRNA gene sequences of pink berry eukaryotes OTUs and cultured relatives. Phylogeny was constructed with FastTree using the approximate maximum-likelihood method (Price *et al*., 2010) and GTRCAT approximation with 20 rate categories. Values at the nodes are SH-like local supports (1000) computed by FastTree and used as condiference values of tree branches. Scale bar represents the number of estimated changes per position for a unit of branch length. **Fig. S4.** Epifluorescence microscopy of CARD-FISH hybridization of sectioned pink berries with a nonsense probe (NON338, panel A), and a *Gammaproteobacteria* probe (GAM42a, panel B). The left side of each panel shows the Alexa 488 probe signal and background autofluorescence; the right side of the panel shows the DAPI stain. **Fig. S5.** Maximum-likelihood phylogeny (FastTree) constructed from an alignment of full-length dsrAB gene sequences (Loy *et al*., 2009). Illumina sequence reads were processed with the Phylosift pipeline which placed them onto this fixed, full-length reference tree using pplacer (Matsen *et al*., 2010). Branches with reads placed are in red, and with a branch line width proportional to the number of assigned sequences. **Fig. S6.** *Chromatiales soxYZ* gene. Generated by RAST synteny viewer using 1e-20 BLASTP similarity cut-off to define and colour gene sets (each set has unique number and colour). Ordered according to % aa similarity to scaffold_341 soxY query. Note the conserved *Chromatiales* hypothetical gene cluster upstream from *soxYZ* (7,12,10,3). **Fig. S7.** *Chromatiales dsrABEFHCMKLJOPNRS* gene cluster is represented in three distinct metagenomic contigs from PB-PSB1. Generated by RAST synteny viewer using 1e-20 BLASTP similarity cut-off to define and colour gene sets (each set has unique number and colour). Ordered according to % aa similarity to scaffold_285 dsrA query. Genes shown in parenthesis at top (*dsrH* and *dsrJ*) were not annotated on metagenomic contigs by RAST but are identifiable as gene fragments using independent BLASTP queries. Also, note the conserved *Chromatiales* hypothetical gene cluster upstream from *dsrAB* (10,17,16/18). **Fig. S8.** Genomic context of *aprM*–*aprBA* gene cluster in PB-PSB1. Generated by RAST synteny viewer using 1e-20 BLASTP similarity cut-off to define and colour gene sets (each set has unique number and colour). Ordered according to % aa similarity to the scaffold_71 aprB query. **Fig. S9.** Genomic context of *aprAB*–*qmoABC* gene cluster in PB-SRB1.Generated by RAST synteny viewer using 1e-20 BLASTP similarity cut-off to define and colour gene sets (each set has unique number and colour). Ordered according to % aa similarity to the scaffold_149 aprA query. **Fig. S10.** Genomic context of *dsrAB* PB-SRB1. Generated by RAST synteny viewer using 1e-20 BLASTP similarity cut-off to define gene sets (each set has unique number and colour). Table below shows annotations for *Desulfobulbaceae*-conserved genes in this genomic region. Ordered according to % aa similarity to scaffold_206 dsrA query. **Fig. S11.** Genomic context of *dsrC* PB-SRB1. Generated by RAST synteny viewer using 1e-20 BLASTP similarity cut-off to define gene sets (each set has unique number and colour). Table below shows annotations for conserved genes in PB-SRB1 in this genomic region. Ordered according to % aa similarity to the scaffold_372 dsrC query. **Fig. S12.** Genomic context of *dsrMKJOP* gene cluster in PB-SRB1. Generated by RAST synteny viewer using 1e-20 BLASTP similarity cut-off to define and colour gene sets (each set has unique number and colour). Ordered according to % aa similarity to the scaffold_12 dsrM query. **Fig. S13.** Elemental analyser–isotope ratio mass spectrometry of pooled berry biomass from dark incubations with either added 34 S-enriched sulfate (‘labelled’) or standard sulfate (‘unlabelled’). Incubations were conducted both with and without 10 mM molybdate. Also shown are three different measurements of pooled berry biomass taken directly from the marsh (‘native’). Values shown are per mil V-CDT, the mean of biological replicates; error bars show standard deviation between these replicates.

Table S1 Quantification of the single copy phylogenetic marker genes for estimating genome completion and number of genomes per bin of the PB-SRB1 and PB-PSB1 associated bins. Note that translation elongation factor EF-2 is duplicated in the closely related *Desulfobulbaceae* genomes. **Table S2.**  Genes of interest in the PB-PSB1 and PB-SBR1 genomes. Gene locations are based on the RAST annotations of the genomes (complete dataset available at FigShare).
