# Supplementary material for: Microscale sulfur cycling in the phototrophic pink berry consortia of the Sippewissett Salt Marsh
Source: Environ Microbiol. 2014 Feb 26;16(11):3398–415. doi: 10.1111/1462-2920.12388 (PMC4262008; doi:10.1111/1462-2920.12388)
Supplement: Supplementary file 1 [file emi0016-3398-SD1.pdf]

Supplemental Results and Discussion:

Metagenomics:

Genome overviews:

The PB-SRB1 bin contained 3.7 Mb of sequence data in 422 contigs greater than 1kb (N50 15kb, max contig 63kb) with 3594 annotated coding sequences. The PB-PSB1 bin contained 6.7 Mb of sequence data in 1,386 contigs greater than 1kb (N50 7.4kb, max contig 31kb) with 6678 annotated coding sequences. Analysis of 45 conserved, single copy marker genes indicated that the PB-SRB1 and PB-PSB1 bins contain near complete genomes (45/45 marker genes) with one phylogenetically coherent copy of each of these genes per bin (Supplemental Table 1). Approximately 73% of annotated coding sequences in the PB-SRB1 draft genome had BLASTp hits (>20% aa identity) to the genomes of cultured relatives, while in the PB-PSB1 genome only 60% of annotated features had hits in sequenced genomes of the *Chromatiaceae*. Annotation details for all genes in the PB-PSB1 and PB-SRB1 genomes discussed in detail below are provided in Supplemental Data.

Oxidative pathway:

We identified homologs of the soluble periplasmic flavocytochrome c (*fccAB*) and two sulfide:quinone oxidoreductases (types IV and VI, homologs of the *sqrD* and *sqrF* genes) implicated in the oxidation of sulfide in the model PSB *Allochromatium vinosum* (Reinartz et al., 1998; Weissgerber and Zigann, 2011). Rhodanese (thiosulfate transferase) genes and the *soxYZ* genes implicated in the utilization of thiosulfate were also present. While related *A. vinosum* possess the *sox* genes in two distinct clusters (*soxYZ, soxBXAKL*) (Weissgerber and Zigann, 2011), only one of these clusters (*soxYZ*) was recovered from the pink berry data (Supplemental Figure 6). Comparison of conserved gene order ('synteny') in the genomes of sequenced sulfur- and sulfide-oxidizing organisms indicates that the organization of the *soxYZ* gene cluster is more widely conserved than is *soxBXAKL* (data not shown).

Reverse-type dissimilatory sulfite reductase (rDsr) proteins are essential for oxidation of stored sulfur to sulfite in *A. vinosum* (Frigaard and Dahl, 2009). Our comparative analysis of publicly available *Chromatiales* genomes indicates that the *rDsr* genes are encoded by a single genomic island, *dsrABEFHCMKLJOPNRS* (Supplemental Figure 7). We recovered these genes from the berry metagenomic data in syntenic arrangement, but spanning three separate scaffolds (Supplemental Figure 7). The oxidation of sulfite to sulfate in purple sulfur bacteria can be mediated by ATP sulfurylase (*sat*) and the membrane-anchored APS reductase complex (*aprMBA* gene cluster); both *sat* and the syntenic *aprMAB* cluster were recovered in genomic contexts similar to those of cultured PSB species (Supplemental Figure 8).

Reductive pathway:

The complete sulfate reduction pathway was found on metagenomic contigs binned to the PB-SRB1 species. The ATP sulfurylase and APS reductase complexes responsible for the reduction of sulfate to sulfite were found in two separate gene clusters in gene contexts consistent with that observed in other *Desulfobulbaceae* genomes (*Desulfocapsa sulfexigens, Desulfobulbus propionicus, Desulfotalea psychrophila*). The *aprBA* operon is located just upstream of the *qmoABC* genes (Supplemental Figure 9), which encode an oxidoreductase complex essential for sulfate reduction (likely via donation of electrons to the AprBA enzyme) (Zane et al., 2010; Pereira et al., 2011). Dsr proteins responsible for the reduction of sulfite to sulfide are encoded in three distinct gene clusters (*dsrABD, dsrC, dsrMKJOP*).

While the *dsr* gene order is broadly conserved among sulfate reducers, the *Desulfobulbaceae* genomes (including the PB-SRB1 metagenomic contigs) share additional gene context unique to this family (Supplemental Figures 10-12).

Possible electron donors for sulfate reduction were investigated by searching for hydrogenases and dehydrogenases in the PB-SRB1 genome. Similar to other SRB genomes (Pereira et al., 2011), the PB-SRB1 genome contained operons with periplasmic uptake Ni-Fe hydrogenase (*hynBAC*), and formate dehydrogenase (*fdhABC*) genes in association with cytochrome-subunits. Furthermore, several other dehydrogenases (lactate, malate, glycine, phytoene, hydroxyisobutyrate, alcohols) were annotated in the PB-SRB1 genome, implying that this organism may be capable of using a variety of organic compounds as electron donors for sulfate reduction. The presence of phytoene dehydrogenases suggests that PB-SRB1 might be able to oxidize photosynthetic pigments in the berries (bacteriochlorophyll, carotenoids). Some of these dehydrogenases (e.g. malate dehydrogenase) likely operate reversibly during gluconeogenesis and carbon storage processes, as the PB-SRB1 genome contains the complete pathways for the TCA cycle, glycolysis, and glycogen metabolism.

The PB-SRB1 genome also encodes the complete Wood–Ljungdahl pathway, which can operate in both the reductive (acetyl-CoA pathway) and oxidative (reverse acetyl-CoA pathway) directions (Thauer et al., 1989; Strittmatter et al., 2009). Operating in the oxidative direction in sulfate-reducing bacteria, the reverse acetyl-CoA pathway catalyzes the complete mineralization of acetyl-CoA to CO_2, generating electrons for the reduction of sulfate. The acetyl-CoA pathway is known to mediate the fixation of CO_2 to cellular biomass during chemolithotrophic growth in the related *Desulfobacterales* species, *Desulfobacterium autotrophicum* HRM2 (Schauder et al., 1988) and *Desulfocapsa sulfexigens* (Frederiksen and Finster, 2004). We observed that the genes encoding this pathway are conserved in all the sequenced genomes from *Desulfobacterales* species for which CO_2-fixation has been reported (*Desulfobacterium autotrophicum* HRM2, *Desulfocapsa sulfexigens*, strain MLMS-1, *Desulfatibacillum alkenivorans* AK-01, *Desulfurivibrio alkaliphilus* AHT2, *Desulfotignum phosphitoxidans* DSM 13687). This finding suggests that the reductive acetyl-CoA pathway is a widely conserved mechanism of CO_2-fixation in the *Desulfobacterales*, and that PB-SRB1 has the genetic potential for both complete acetate oxidation and facultative autotrophy via this pathway.

The mechanisms controlling the switch from oxidative to reductive operation of this pathway remain unclear. In PB-SRB1, an intriguing regulatory mechanism was suggested by the discovery of a homolog to the hybrid-cluster protein regulator gene (*hcpR*) in the region upstream of the carbon monoxide dehydrogenase operon (*cdhACED*), which encodes key steps in the Wood–Ljungdahl pathway. *hcpR*, a member of the Crp/Fnr family of transcriptional regulators, is predicted to be a transcriptional activator of the oxidative carbon monoxide dehydrogenase operon in *Desulfovibrio* species (Rodionov et al., 2004), suggesting that this protein may also serve to regulate the *cdhACED* genes within PB-SRB1.

<u>Microvoltammetry</u>:

While most consistent with sulfide, the signal referenced at 0.85 V (vs. Ag/AgCl) could also be due to the presence of polysulfide in the aggregate, as the pH was not specifically measured for this microenvironment. Previous investigations have reported a pH range from 8.2 at the surface of a berry to 7.9 at the interior (Seitz et al., 1993). Given that it is unusual for polysulfide to form in the absence of sulfide at a pH below 9 (Kamyshny et al., 2004) and that only a single peak was observed, it is most likely that the signal observed was from sulfide.

Other berries similarly probed with voltammetric electrodes did not reveal this measurable sulfide peak, with detection limits for sulfide in these waters of less than 0.1 μM (Luther et al., 2008). This could be due to either inherent differences in metabolic activity between berries, or the loss of sulfide by diffusion, degassing, or oxidation before electrochemical measurements could be made. No other electroactive species detectable by cyclic voltammetry (e.g. O_2, Fe^{3+}, Fe^{2+}, Mn^{2+}, and As^{3+}, tetrathionate, dissolved/nanoparticulate elemental sulfur) were observed within the berries. Although elemental sulfur deposits in PB-PSB1 were evident by microscopy (Figure 3), we could not detect this pool of elemental sulfur using microvoltammetry, likely because these inclusions were insulated from the electrode both by their protein coat and their location inside the cell.

Sulfur isotopic fractionation:

The range of sulfide-sulfate fractionations observed in the berries (15‰ at the periphery to 53‰ at the aggregate center) is consistent with values reported from pure cultures of sulfate reducing bacteria which can range from ~0‰ to 66‰ (Sim et al., 2011a). Large isotopic fractionations, such as those observed in the center of the berries, can be produced by slow-growing sulfate reducing bacteria cultured with refractory or growth-limiting concentrations of electron donors (Sim et al., 2011a; Sim et al., 2011b; Leavitt et al., 2013). The observed isotopic gradients could thus be explained solely by changes in the supply or identity of electron donors for sulfate reduction, with slower rates of sulfate reduction occurring towards the center of the aggregate, away from the redox gradients at the berry perimeters. The possibility that changes in the availability of electron donors alone could produce this isotopic variation indicates that more careful studies of carbon flux and sulfate reduction rates are necessary to further our understanding in this area.

The large fractionations observed at the center of the berry could also be produced in a step-wise fashion from the coupled metabolic activities of reductive and oxidative processes. As sulfide oxidation has little isotopic effect, PB-PSB1's re-oxidation of syntrophically supplied, isotopically depleted sulfide would generate a local pool of isotopically-depleted sulfate and elemental sulfur (Figure 9). Successive rounds of either sulfate reduction or sulfur disproportionation by PB-SRB1 could progressively increase the total fractionation between produced sulfide and original seawater sulfate. This process would be expected to have the largest impact towards the center of the aggregate, where mixing with seawater sulfate would be minimized by diffusional barriers (Petroff et al., 2011). When coupled with sulfate reduction, the disproportionation of elemental sulfur is known to produce large isotopic fractionations, and, until recently, had been the standard explanation for observations of sulfide-sulfate fractionations in excess of 46‰ (e.g. Canfield and Teske, 1996).

Supplemental Experimental Procedures:

PCR amplification, cloning, Sanger capillary sequencing, and analysis

Bacterial 16S rRNA genes were amplified with the Promega 2x MasterMix using the 8F and 1492R primers (Lane, 1991) at a final concentration of 0.2 μM. PCR was performed at 95°C for 5 minutes followed by 30 cycles of 95°C for 30 seconds, 46°C for 30 seconds and 72°C for 1.5 minutes and a final 72°C extension for 5 minutes. Eukaryotic 18S rRNA genes were amplified with the 360FE and 1492R primer pair as described previously (Dawson and Pace, 2002). All PCR products were gel purified with the Qiaquick Gel Extraction kit (Qiagen Corp., Valencia, CA) and cloned using the One Shot TOP10

electrocompetent cells (Invitrogen). Colonies (96) from each sample were selected randomly and sequenced unidirectionally using the 8F primer. Capillary sequencing was performed using the Applied Biosystems model 3730 Sanger sequencer at the Marine Biological Laboratory's Josephine Bay Paul Center.

Bases were called from each chromatogram using Phred (Ewing and Green, 1998; Ewing et al., 1998). Sequences were quality trimmed as described previously (Pepe-Ranney et al., 2012), filtered with sequences below 400 bp in length discarded from future analysis. Chimera checking was performed with UCHIME (Edgar et al., 2011). Select clones from phylotypes of interest were sequenced bidirectionally and assembled using Phrap (Phil Green, http://www.phrap.org). Sequences were clustered into operational taxonomic units (OTUs) at the 97% sequence similarity threshold using UCLUST (Edgar, 2010) as implemented in the QIIME pipeline (Caporaso et al., 2010). SSU rRNA gene sequences were aligned to the SILVA 108 database (Pruesse et al., 2007) using SINA (Pruesse et al., 2012) and curated using ARB (Ludwig et al., 2004).

Metagenomic sequencing

Roche GS Titanium 454 sequencing was performed at the Pennsylvania State University genomics facility, generating 82 Mb of sequence data (220,000 reads with an average length of 360bp). Illumina library preparation and sequencing following standard protocols was carried out at the UC Davis DNA technologies Core Facility (http://dnatech.genomecenter.ucdavis.edu/). Illumina libraries were prepared using Illumina's Nextera DNA Sample Preparation kit. Samples sequenced with the Illumina HiSeq were barcoded with several unrelated samples in one lane, generating ~600 Mb of pink berry sequence data from 100 bp paired-end (PE) reads. Illumina MiSeq sequencing was conducted using the newly available 250 bp PE sequencing chemistry to generate a large dataset (25 million) of long reads suitable for assessments of microbial diversity from unassembled sequence data. Reads were quality filtered and trimmed using the FASTX toolkit (http://hannonlab.cshl.edu/fastx_toolkit/). Sequencing primer and adapter contamination were filtered using the TagDust package (Lassmann et al., 2009).

Metagenomic assembly, binning and genome completeness

Contigs larger than 1 kb were binned by tetranucleotide frequencies using an emergent self-organized map (ESOMs, 3kb TNF window, 100 training epochs, 147 rows x 240 columns), as described previously (Dick et al., 2009; Wrighton et al., 2012). Bins apparent in the TNF ESOM were further refined using sequence coverage information (Wrighton et al., 2012). Genomic bins apparent in the ESOM were assigned to organisms using best BLAST-based binning (Huson et al., 2011) and phylogenetic marker genes (Darling et al., submitted). Draft genomes for the pink berry purple sulfur bacteria (PB-PSB1) and sulfate reducing bacteria (PB-SRB1) species were annotated using the RAST server (Aziz et al., 2008) and SEED database (Overbeek et al., 2005). Genome completeness was assessed using 45 conserved, single copy marker genes (Raes et al., 2007; Wrighton et al., 2012; Wu et al., 2013).

CARD-FISH

In addition to hybridizations with the custom SRB-PiBe213 probe, GAM42A (Manz et al., 1992) and Delta495a-c with competitors a-c (Lücker et al., 2007), the eubacterial probe EUB338I-III (Amann et al., 1990; Daims et al., 1999) was used as a positive control and a nonsense probe NON338 (Wallner et al., 1993) as a control for nonspecific binding. Hybridizations and tyramide signal amplification was performed as described previously (Ishii et al., 2004) with the following modifications. All ethanol dehydrations were omitted and tissue sections were permeabilized with lysozyme (10 mg/mL in PBS) for

1 hour at 37°C followed by Triton X-100 (0.05% in PBS) for 2-5 minutes. Internal peroxidases were inactivated with 0.01 M HCl for 20 minutes at room temperature and air dried. Hybridizations were conducted at final probe concentration 0.17 ng/µl at 46°C for 3 hours, and subsequently incubated 15 minutes in prewarmed wash buffer at 48°C. Tyramide signal amplification was performed for two hours at 46°C with an Alexa488-tyramide conjugate (1 mg dye/mL). Tissue sections were mounted in ProLong® Gold Antifade Reagent with DAPI (Invitrogen) and stored at -20°C prior to analysis. For the SRB-PiBE213 probe, hybridizations were performed with 10, 20, 30, 40, 45 and 50% formamide in the hybridization buffer. Signals at 50% formamide were not distinguishable from background autofluorescence of the aggregate, but the 45% formamide hybridization revealed clear and intense signals of the rod-shaped PB-SRB1 cells. Subsequent hybridizations were conducted at 45% formamide.

Sulfide capture and SIMS 7f-Geo ion microprobe analysis of $\delta^{34}S$

A transect through two consecutive berry sulfide films was conducted in one continuous chained analysis without instrument retuning. For each spot, the primary beam was rastered over a 25 µm by 25 µm area, while mass 32 and 34 ions were collected using the electron multiplier (counts per second on mass 32 were approximately 500,000 near the berry interior). Successive spots in the transect were spaced 75 µm apart. Instrumental mass fractionation was corrected for by standard-sample bracketing throughout the transect, where the standard was a similar 24 gauge silver wire exposed to a sodium sulfide solution of known isotopic composition ($\delta^{34}S$ = 14.75‰), resulting in the precipitation of silver sulfide on the wire with approximately the same concentrations as the berry interiors (peak yield on mass 32 of ~ 5e5 to 1e6 counts/second). Using these bracketed controls, we assessed the impact of instrument drift over the $\delta^{34}S$ over the course of this transect and found it to be ~1‰, comparable to measurement precision and much smaller than the ~35‰ gradients observed between berry edges and centers. Sulfur isotope values are reported in per mil (‰) relative to the V-CDT (Vienna Canyon Diablo Troilite) scale.

Supplemental References:

Amann, R.I., Binder, B.J., Olson, R.J., Chisholm, S.W., Devereux, R., and Stahl, D.A. (1990) Combination of 16S rRNA-targeted oligonucleotide probes with flow cytometry for analyzing mixed microbial populations. *Applied and environmental microbiology* **56**: 1919-1925.

Aziz, R.K., Bartels, D., Best, A.A., DeJongh, M., Disz, T., Edwards, R.A. et al. (2008) The RAST Server: rapid annotations using subsystems technology. *BMC Genomics* **9**: 75.

Canfield, D., and Teske, A. (1996) Late Proterozoic rise in atmospheric oxygen concentration inferred from phylogenetic and sulphur-isotope studies. *Nature* **382**: 127-132.

Caporaso, J.G., Kuczynski, J., Stombaugh, J., Bittinger, K., Bushman, F.D., Costello, E.K. et al. (2010) QIIME allows analysis of high-throughput community sequencing data. *Nat Methods* **7**: 335-336.

Daims, H., Brühl, A., Amann, R., Schleifer, K.-H., and Wagner, M. (1999) The Domain-specific Probe EUB338 is Insufficient for the Detection of all Bacteria: Development and Evaluation of a more Comprehensive Probe Set. *Systematic and Applied Microbiology* **22**: 434-444.

Darling, A.E., Jospin, G., Bik, H., and Eisen, J.A. (submitted) Phylosift: metagenome phylogeny for the masses.

Dawson, S.C., and Pace, N.R. (2002) Novel kingdom-level eukaryotic diversity in anoxic environments. *Proc Natl Acad Sci U S A* **99**: 8324-8329.

Dick, G.J., Andersson, A.F., Baker, B.J., Simmons, S.L., Thomas, B.C., Yelton, a.P., and Banfield, J.F. (2009) Community-wide analysis of microbial genome sequence signatures. *Genome Biology* **10**: R85-R85.

Edgar, R.C. (2010) Search and clustering orders of magnitude faster than BLAST. *Bioinformatics* **26**: 2460-2461.

Edgar, R.C., Haas, B.J., Clemente, J.C., Quince, C., and Knight, R. (2011) UCHIME improves sensitivity and speed of chimera detection. *Bioinformatics* **27**: 2194-2200.

Ewing, B., and Green, P. (1998) Base-calling of automated sequencer traces using phred. II. Error probabilities. *Genome Res* **8**: 186-194.

Ewing, B., Hillier, L., Wendl, M.C., and Green, P. (1998) Base-calling of automated sequencer traces using phred. I. Accuracy assessment. *Genome Res* **8**: 175-185.

Frederiksen, T.-M., and Finster, K. (2004) The transformation of inorganic sulfur compounds and the assimilation of organic and inorganic carbon by the sulfur disproportionating bacterium *Desulfocapsa sulfoexigens*. *Antonie van Leeuwenhoek* **85**: 141-149.

Frigaard, N.-U., and Dahl, C. (2009) *Sulfur metabolism in phototrophic sulfur bacteria*.

Huson, D.H., Mitra, S., Ruscheweyh, H.J., Weber, N., and Schuster, S.C. (2011) Integrative analysis of environmental sequences using MEGAN4. *Genome Res* **21**: 1552-1560.

Ishii, K., Mussmann, M., MacGregor, B.J., and Amann, R. (2004) An improved fluorescence in situ hybridization protocol for the identification of bacteria and archaea in marine sediments. *FEMS Microbiol Ecol* **50**: 203-213.

Kamyshny, A., Goifman, A., Gun, J., Rizkov, D., and Lev, O. (2004) Equilibrium distribution of polysulfide ions in aqueous solutions at 25C: A new approach for the study of polysulfides' equilibria. *Environmental Science & Technology* **38**: 6633-6644.

Lane, D. (1991) 16S/23S rRNA sequencing. In *Nucleic Acid Techniques in Bacteria Systematics*. Stackebrandt, E., and Goodfellow, M. (eds). Chichester, UK: Wiley and Sons, p. 329.

Lassmann, T., Hayashizaki, Y., and Daub, C.O. (2009) TagDust--a program to eliminate artifacts from next generation sequencing data. *Bioinformatics* **25**: 2839-2840.

Leavitt, W.D., Halevy, I., Bradley, A.S., and Johnston, D.T. (2013) Influence of sulfate reduction rates on the Phanerozoic sulfur isotope record. *Proc Natl Acad Sci U S A*.

Lücker, S., Steger, D., Kjeldsen, K.U., MacGregor, B.J., Wagner, M., and Loy, A. (2007) Improved 16S rRNA-targeted probe set for analysis of sulfate-reducing bacteria by fluorescence in situ hybridization. *Journal of microbiological methods* **69**: 523-528.

Ludwig, W., Strunk, O., Westram, R., Richter, L., Meier, H., Yadhukumar et al. (2004) ARB: a software environment for sequence data. *Nucleic Acids Res* **32**: 1363-1371.

Luther, G.W., III, Glazer, B.T., Ma, S., Trouwborst, R.E., Moore, T.S., Metzger, E. et al. (2008) Use of voltammetric solid-state (micro)electrodes for studying biogeochemical processes: Laboratory measurements to real time measurements with an in situ electrochemical analyzer (ISEA). *Marine Chemistry* **108**: 221-235.

Manz, W., Amann, R., Ludwig, W., Wagner, M., and Schleifer, K.-H. (1992) Phylogenetic Oligodeoxynucleotide Probes for the Major Subclasses of Proteobacteria: Problems and Solutions. *Systematic and Applied Microbiology* **15**: 593-600.

Overbeek, R., Begley, T., Butler, R.M., Choudhuri, J.V., Chuang, H.Y., Cohoon, M. et al. (2005) The subsystems approach to genome annotation and its use in the project to annotate 1000 genomes. *Nucleic Acids Res* **33**: 5691-5702.

Pepe-Ranney, C., Berelson, W.M., Corsetti, F.A., Treants, M., and Spear, J.R. (2012) Cyanobacterial construction of hot spring siliceous stromatolites in Yellowstone National Park. *Environ Microbiol* **14**: 1182-1197.

Pereira, I.A., Ramos, A.R., Grein, F., Marques, M.C., da Silva, S.M., and Venceslau, S.S. (2011) A comparative genomic analysis of energy metabolism in sulfate reducing bacteria and archaea. *Front Microbiol* **2**: 69.

Petroff, A.P., Wu, T.D., Liang, B., Mui, J., Guerquin-Kern, J.L., Vali, H. et al. (2011) Reaction-diffusion model of nutrient uptake in a biofilm: theory and experiment. *J Theor Biol* **289**: 90-95.

Pruesse, E., Peplies, J., and Glockner, F.O. (2012) SINA: accurate high-throughput multiple sequence alignment of ribosomal RNA genes. *Bioinformatics* **28**: 1823-1829.

Pruesse, E., Quast, C., Knittel, K., Fuchs, B.M., Ludwig, W., Peplies, J., and Glockner, F.O. (2007) SILVA: a comprehensive online resource for quality checked and aligned ribosomal RNA sequence data compatible with ARB. *Nucleic Acids Res* **35**: 7188-7196.

Raes, J., Korbel, J.O., Lercher, M.J., von Mering, C., and Bork, P. (2007) Prediction of effective genome size in metagenomic samples. *Genome biology* **8**: R10-R10.

Reinartz, M., Tschape, J., Bruser, T., Truper, H.G., and Dahl, C. (1998) Sulfide oxidation in the phototrophic sulfur bacterium Chromatium vinosum. *Arch Microbiol* **170**: 59-68.

Rodionov, D.A., Dubchak, I., Arkin, A., Alm, E., and Gelfand, M.S. (2004) Reconstruction of regulatory and metabolic pathways in metal-reducing delta-proteobacteria. *Genome Biol* **5**: R90.

Schauder, R., Preuß, A., Jetten, M., and Fuchs, G. (1988) Oxidative and reductive acetyl CoA/carbon monoxide dehydrogenase pathway in Desulfobacterium autotrophicum. *Arch Microbiol* **151**: 84-89.

Seitz, A.P., Nielsen, T.H., and Overmann, J. (1993) Physiology of purple sulfur bacteria forming macroscopic aggregates in Great Sippewissett Salt Marsh, Massachusetts. *FEMS Microbiology Ecology* **12**: 225-235.

Sim, M.S., Bosak, T., and Ono, S. (2011a) Large sulfur isotope fractionation does not require disproportionation. *Science* **333**: 74-77.

Sim, M.S., Ono, S., Donovan, K., Templer, S.P., and Bosak, T. (2011b) Effect of electron donors on the fractionation of sulfur isotopes by a marine Desulfovibrio sp. *Geochimica et Cosmochimica Acta* **75**: 4244-4259.

Strittmatter, A.W., Liesegang, H., Rabus, R., Decker, I., Amann, J., Andres, S. et al. (2009) Genome sequence of Desulfobacterium autotrophicum HRM2, a marine sulfate reducer oxidizing organic carbon completely to carbon dioxide. *Environ Microbiol* **11**: 1038-1055.

Thauer, R.K., Moller-Zinkhan, D., and Spormann, A.M. (1989) Biochemistry of acetate catabolism in anaerobic chemotrophic bacteria. *Annu Rev Microbiol* **43**: 43-67.

Wallner, G., Amann, R., and Beisker, W. (1993) Optimizing fluorescent in situ hybridization with rRNA-targeted oligonucleotide probes for flow cytometric identification of microorganisms. *Cytometry* **14**: 136-143.

Weissgerber, T., and Zigann, R. (2011) Complete genome sequence of Allochromatium vinosum DSM 180 T. *Standards in …*: 311-330.

Wrighton, K.C., Thomas, B.C., Sharon, I., Miller, C.S., Castelle, C.J., VerBerkmoes, N.C. et al. (2012) Fermentation, hydrogen, and sulfur metabolism in multiple uncultivated bacterial phyla. *Science* **337**: 1661-1665.

Wu, D., Jospin, G., and Eisen, J.A. (2013) Systematic Identification of Gene Families for Use as “Markers” for Phylogenetic and Phylogeny-Driven Ecological Studies of Bacteria and Archaea and Their Major Subgroups. *PLoS ONE* **8**: e77033.

Zane, G.M., Yen, H.C., and Wall, J.D. (2010) Effect of the deletion of qmoABC and the promoter-distal gene encoding a hypothetical protein on sulfate reduction in Desulfovibrio vulgaris Hildenborough. *Appl Environ Microbiol* **76**: 5500-5509.
