# Supplementary material for: Microscale sulfur cycling in the phototrophic pink berry consortia of the Sippewissett Salt Marsh
Source: Environ Microbiol. 2014 Feb 26;16(11):3398–415. doi: 10.1111/1462-2920.12388 (PMC4262008; doi:10.1111/1462-2920.12388)
Supplement: Table S1 — Quantification of the single copy phylogenetic marker genes for estimating genome completion and number of genomes per bin of the PB-SRB1 and PB-PSB1 associated bins. Note that translation elongation factor EF-2 is duplicated in the closely related Desulfobulbaceae genomes. Table S2. Genes of interest in the PB-PSB1 and PB-SBR1 genomes. Gene locations are based on the RAST annotations of the genomes (complete dataset available at FigShare). [file emi0016-3398-SD4.pdf]

Supplemental Table 1. Quantification of the single copy phylogenetic marker genes for estimating genome completion and number of genomes per bin of the PB-SRB1 and PB-PSB1 associated bins. Note that translation elongation factor EF-2 is duplicated in the closely related *Desulfobulbaceae* genomes

| Conserved single-copy marker gene | PB-SRB1 | PB-PSB1 |
|---:|---|---|
| recA | 1 | 1 |
| RpoB | 1 | 1 |
| DNA gyrase A | 1 | 1 |
| DNA gyrase B | 1 | 1 |
| ffh signal recognition particle protein | 1 | 1 |
| Chaperone protein HtpG | 1 | 1 |
| YgjD/Kae1/Qri7 family metalloendopeptidase | 1 | 1 |
| phosphoribosylformylglycinamidine cyclo-ligase | 1 | 1 |
| Porphobilinogen deaminase | 1 | 1 |
| ribonuclease HII | 1 | 1 |
| phenylalanyl-tRNA synthetase alpha subunit | 1 | 1 |
| phenylalanyl-tRNA synthetase beta subunit | 1 | 1 |
| tRNA pseudouridine synthase B | 1 | 1 |
| translation elongation factor EF-2 | 2 | 1 |
| translation initiation factor IF-2 | 1 | 1 |
| ribosomal protein S2 | 1 | 1 |
| ribosomal protein S3 | 1 | 1 |
| ribosomal protein S5 | 1 | 1 |
| ribosomal protein S7 | 1 | 1 |
| ribosomal protein S8 | 1 | 1 |
| ribosomal protein S9 | 1 | 1 |
| ribosomal protein S10 | 1 | 1 |
| ribosomal protein S11 | 1 | 1 |
| ribosomal protein S12/S23 | 1 | 1 |
| ribosomal protein S13 | 1 | 1 |
| ribosomal protein S15P | 1 | 1 |
| ribosomal protein S17 | 1 | 1 |
| ribosomal protein S19 | 1 | 1 |
| ribosomal protein L1 | 1 | 1 |
| ribosomal protein L2 | 1 | 1 |
| ribosomal protein L3 | 1 | 1 |
| ribosomal protein L4/L1e | 1 | 1 |
| ribosomal protein L5 | 1 | 1 |
| ribosomal protein L6 | 1 | 1 |
| ribosomal protein L10 | 1 | 1 |
| ribosomal protein L11 | 1 | 1 |
| ribosomal protein L13 | 1 | 1 |
| ribosomal protein L14b | 1 | 1 |
| ribosomal protein L15 | 1 | 1 |
| ribosomal protein L16 | 1 | 1 |
| ribosomal protein L18P/L5E | 1 | 1 |
| ribosomal protein L22 | 1 | 1 |
| ribosomal protein L24 | 1 | 1 |
| ribosomal protein L25/L23 | 1 | 1 |
| ribosomal protein L29 | 1 | 1 |

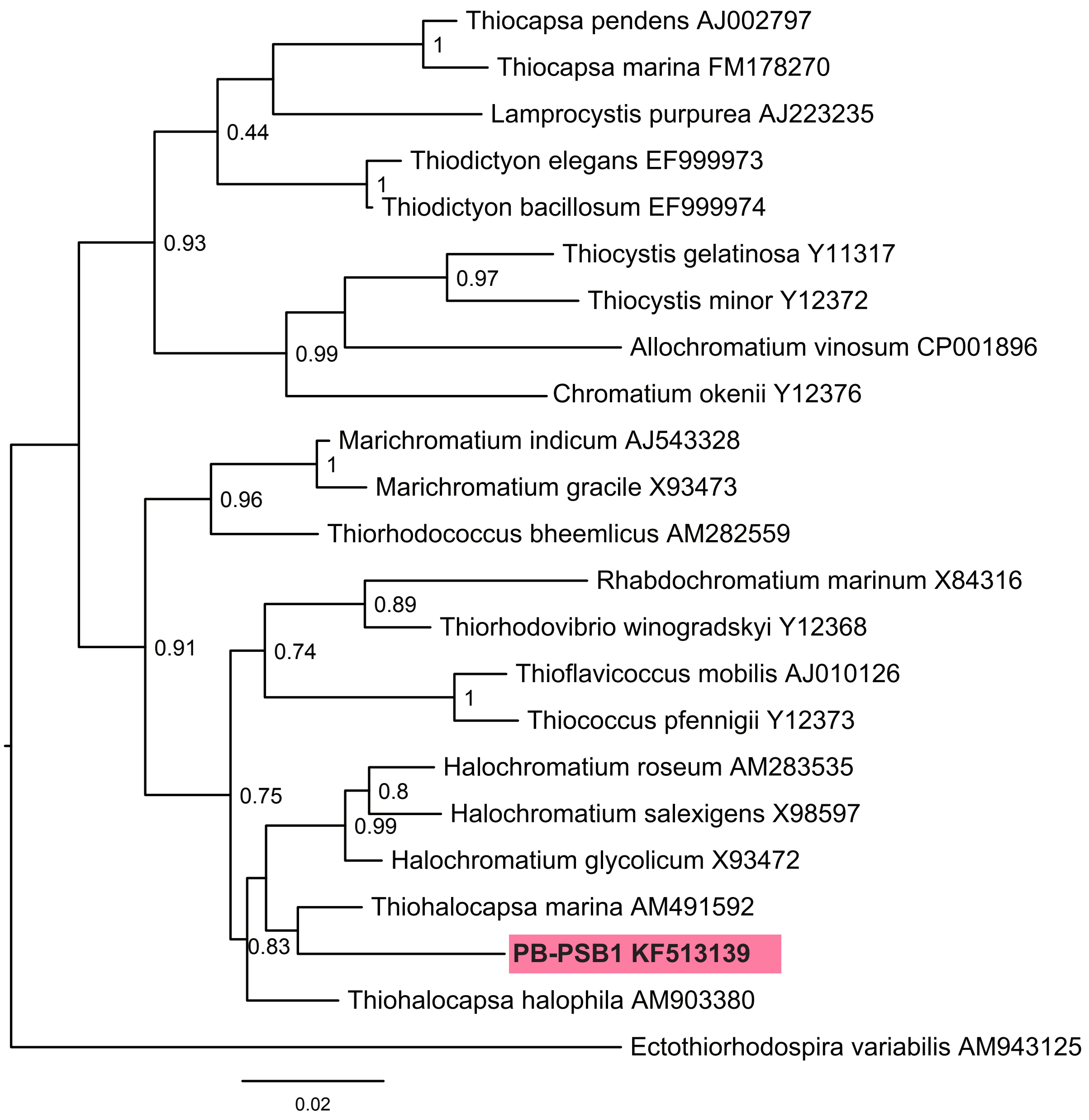


**Supplemental Figure 1.** Maximum likelihood phylogeny (rooted) constructed from 16S rRNA gene sequences of pink berry purple sulfur bacteria PB-PSB1 and cultured *Chromatiales* relatives. Phylogeny was constructed with FastTree using the approximate maximum likelihood method (Price et al., 2010) and GTRCAT approximation with 20 rate categories. Values at the nodes are SH-like local supports (1000) computed by FastTree and used as condiference values of tree branches. Scale bar represents the number of estimated changes per position for a unit of branch length.

**Supplemental Figure 2.** Maximum likelihood phylogeny (mid-point rooted) constructed from 16S rRNA gene sequences of pink berry Bacteroidetes phylotypes OTUs (pink), cultured isolates (blue), and uncultured environmental amplicon. Phylogeny was constructed with FastTree using the approximate maximum likelihood method (Price et al., 2010) and GTRCAT approximation with 20 rate categories. Values at the nodes are SH-like local supports (1000) computed by FastTree and used as condiference values of tree branches. Scale bar represents the number of estimated changes per position for a unit of branch length. Marked with red stars are the dominant pink berry phylotypes. Note that the relative scarcity of cultured isolates and abundance of related sequences from diverse sulfur-cycling habitats

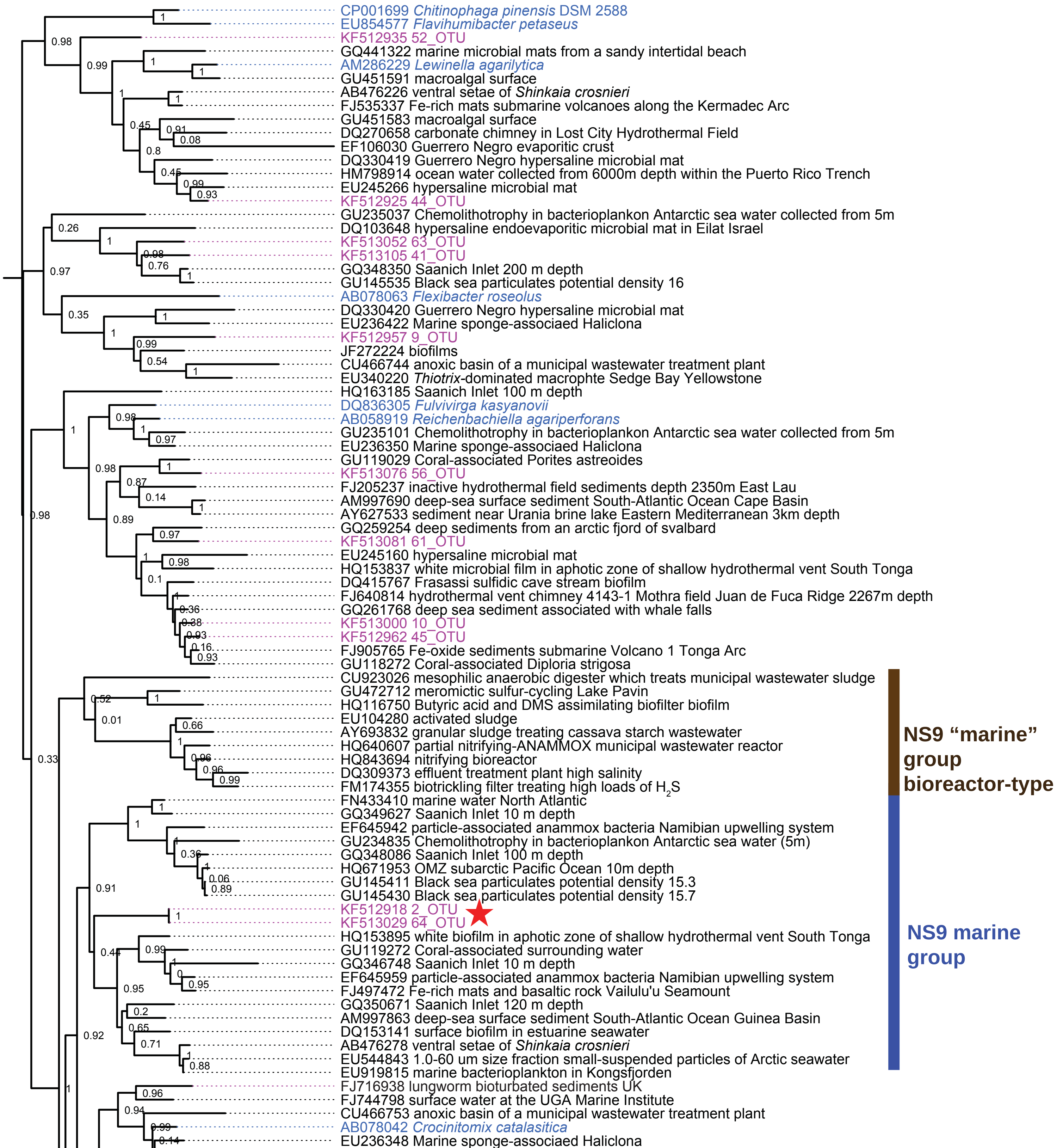

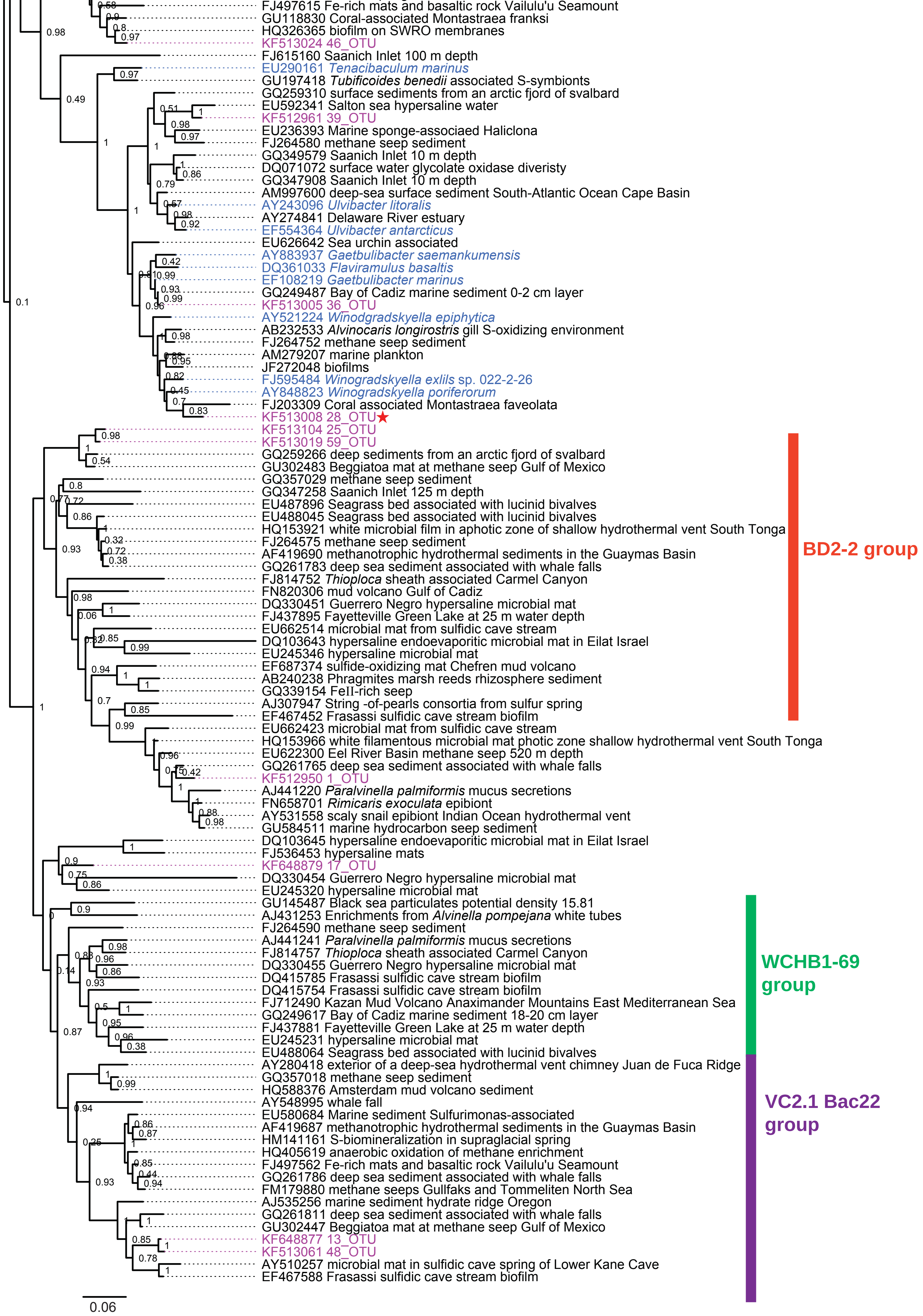
FJ497615 Fe-rich mats and basaltic rock Vailulu'u Seamount
GU118830 Coral-associated Montastraea franksi
HQ326365 biofilm on SWRO membranes
KF513024 46_OTU
FJ615160 Saanich Inlet 100 m depth
EU290161 Tenacibaculum marinus
GU197418 Tubificoides benedii associated S-symbionts
GQ259310 surface sediments from an arctic fjord of svalbard
EU592341 Salton sea hypersaline water
KF512961 39_OTU
EU236393 Marine sponge-associaed Haliclona
FJ264580 methane seep sediment
GQ349579 Saanich Inlet 10 m depth
DQ071072 surface water glycolate oxidase diveristy
GQ347908 Saanich Inlet 10 m depth
AM997600 deep-sea surface sediment South-Atlantic Ocean Cape Basin
AY243096 Ulvibacter litoralis
AY274841 Delaware River estuary
EF554364 Ulvibacter antarcticus
EU626642 Sea urchin associated
AY883937 Gaetbulibacter saemankumensis
DQ361033 Flaviramulus basaltis
EF108219 Gaetbulibacter marinus
GQ249487 Bay of Cadiz marine sediment 0-2 cm layer
KF513005 36_OTU
AY521224 Winodgradskyella epiphytica
AB232533 Alvinocaris longirostris gill S-oxidizing environment
FJ264752 methane seep sediment
AM279207 marine plankton
JF272048 biofilms
FJ595484 Winogradskyella exlils sp. 022-2-26
AY848823 Winogradskyella poriferorum
FJ203309 Coral associated Montastraea faveolata
KF513008 28_OTU★
KF513104 25_OTU
KF513019 59_OTU
GQ259266 deep sediments from an arctic fjord of svalbard
GU302483 Beggiatoa mat at methane seep Gulf of Mexico
GQ357029 methane seep sediment
GQ347258 Saanich Inlet 125 m depth
EU487896 Seagrass bed associated with lucinid bivalves
EU488045 Seagrass bed associated with lucinid bivalves
HQ153921 white microbial film in aphotic zone of shallow hydrothermal vent South Tonga
FJ264575 methane seep sediment
AF419690 methanotrophic hydrothermal sediments in the Guaymas Basin
GQ261783 deep sea sediment associated with whale falls
FJ814752 Thioploca sheath associated Carmel Canyon
FN820306 mud volcano Gulf of Cadiz
DQ330451 Guerrero Negro hypersaline microbial mat
FJ437895 Fayetteville Green Lake at 25 m water depth
EU662514 microbial mat from sulfidic cave stream
DQ103643 hypersaline endoevaporitic microbial mat in Eilat Israel
EU245346 hypersaline microbial mat
EF687374 sulfide-oxidizing mat Chefren mud volcano
AB240238 Phragmites marsh reeds rhizosphere sediment
GQ339154 FeII-rich seep
AJ307947 String -of-pearls consortia from sulfur spring
EF467452 Frasassi sulfidic cave stream biofilm
EU662423 microbial mat from sulfidic cave stream
HQ153966 white filamentous microbial mat photic zone shallow hydrothermal vent South Tonga
EU622300 Eel River Basin methane seep 520 m depth
GQ261765 deep sea sediment associated with whale falls
KF512950 1_OTU
AJ441220 Paralvinella palmiformis mucus secretions
FN658701 Rimicaris exoculata epibiont
AY531558 scaly snail epibiont Indian Ocean hydrothermal vent
GU584511 marine hydrocarbon seep sediment
DQ103645 hypersaline endoevaporitic microbial mat in Eilat Israel
FJ536453 hypersaline mats
KF648879 17_OTU
DQ330454 Guerrero Negro hypersaline microbial mat
EU245320 hypersaline microbial mat
GU145487 Black sea particulates potential density 15.81
AJ431253 Enrichments from Alvinella pompejana white tubes
FJ264590 methane seep sediment
AJ441241 Paralvinella palmiformis mucus secretions
FJ814757 Thioploca sheath associated Carmel Canyon
DQ330455 Guerrero Negro hypersaline microbial mat
DQ415785 Frasassi sulfidic cave stream biofilm
DQ415754 Frasassi sulfidic cave stream biofilm
FJ712490 Kazan Mud Volcano Anaximander Mountains East Mediterranean Sea
GQ249617 Bay of Cadiz marine sediment 18-20 cm layer
FJ437881 Fayetteville Green Lake at 25 m water depth
EU245231 hypersaline microbial mat
EU488064 Seagrass bed associated with lucinid bivalves
AY280418 exterior of a deep-sea hydrothermal vent chimney Juan de Fuca Ridge
GQ357018 methane seep sediment
HQ588376 Amsterdam mud volcano sediment
AY548995 whale fall
EU580684 Marine sediment Sulfurimonas-associated
AF419687 methanotrophic hydrothermal sediments in the Guaymas Basin
HM141161 S-biomineralization in supraglacial spring
HQ405619 anaerobic oxidation of methane enrichment
FJ497562 Fe-rich mats and basaltic rock Vailulu'u Seamount
GQ261786 deep sea sediment associated with whale falls
FM179880 methane seeps Gullfaks and Tommeliten North Sea
AJ535256 marine sediment hydrate ridge Oregon
GQ261811 deep sea sediment associated with whale falls
GU302447 Beggiatoa mat at methane seep Gulf of Mexico
KF648877 13_OTU
KF513061 48_OTU
AY510257 microbial mat in sulfidic cave spring of Lower Kane Cave
EF467588 Frasassi sulfidic cave stream biofilm
BD2-2 group
WCHB1-69 group
VC2.1 Bac22 group
0.06

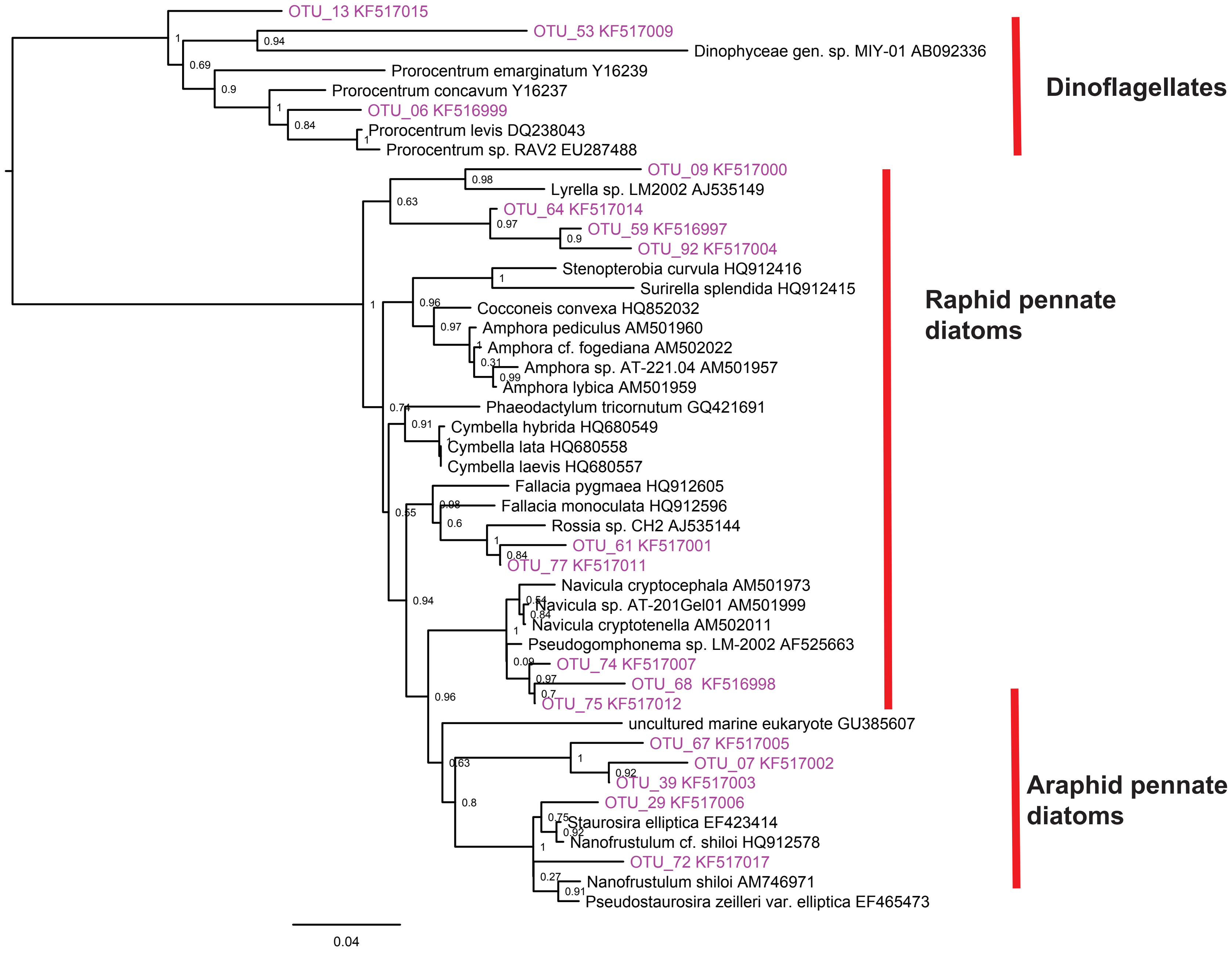


**Supplemental Figure 3.** Maximum likelihood phylogeny (mid-point rooted) constructed from 18S rRNA gene sequences of pink berry eukaryotes OTUs and cultured relatives. Phylogeny was constructed with FastTree using the approximate maximum likelihood method (Price et al., 2010) and GTRCAT approximation with 20 rate categories. Values at the nodes are SH-like local supports (1000) computed by FastTree and used as condiference values of tree branches. Scale bar represents the number of estimated changes per position for a unit of branch length.

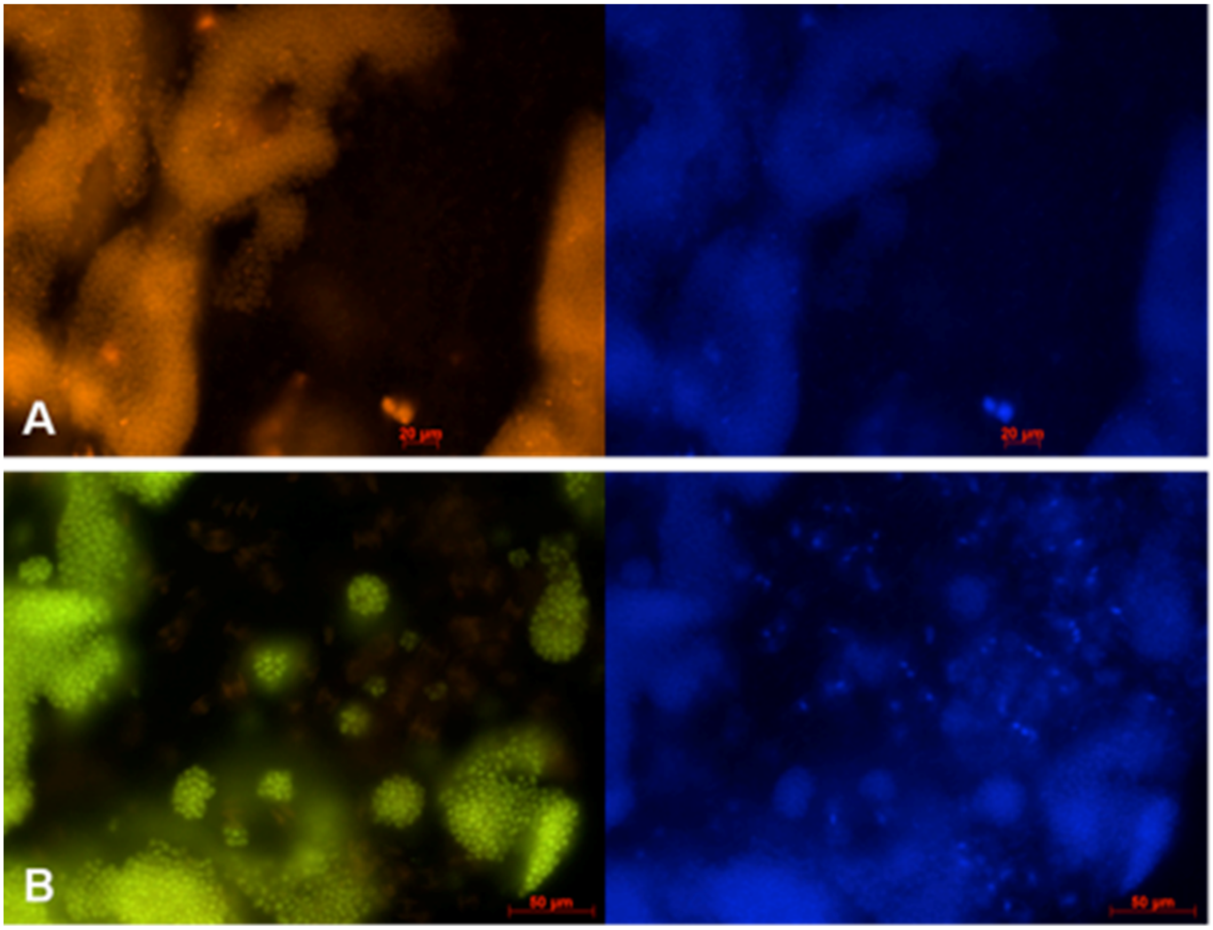


**Supplemental Figure 4.** Epifluorescence microscopy of CARD-FISH hybridization of sectioned pink berries with a nonsense probe (NON338, panel **A**), and a *Gammaproteobacteria* probe (GAM42a, panel **B**). The left side of each panel shows the Alexa 488 probe signal and background autofluorescence, the right side of the panel shows the DAPI stain.

**Supplemental Figure 5.** Maximum likelihood phylogeny (FastTree) constructed from an alignment of full length *dsrAB* gene sequences (Loy et al., 2009). Illumina sequence reads were processed withthe Phylosift pipeline which placed them onto this fixed, full length reference tree using pplacer (Matsen et al., 2010). Branches with reads placed are in red, and with a branch line width proportional to the number of assigned seqeunces.

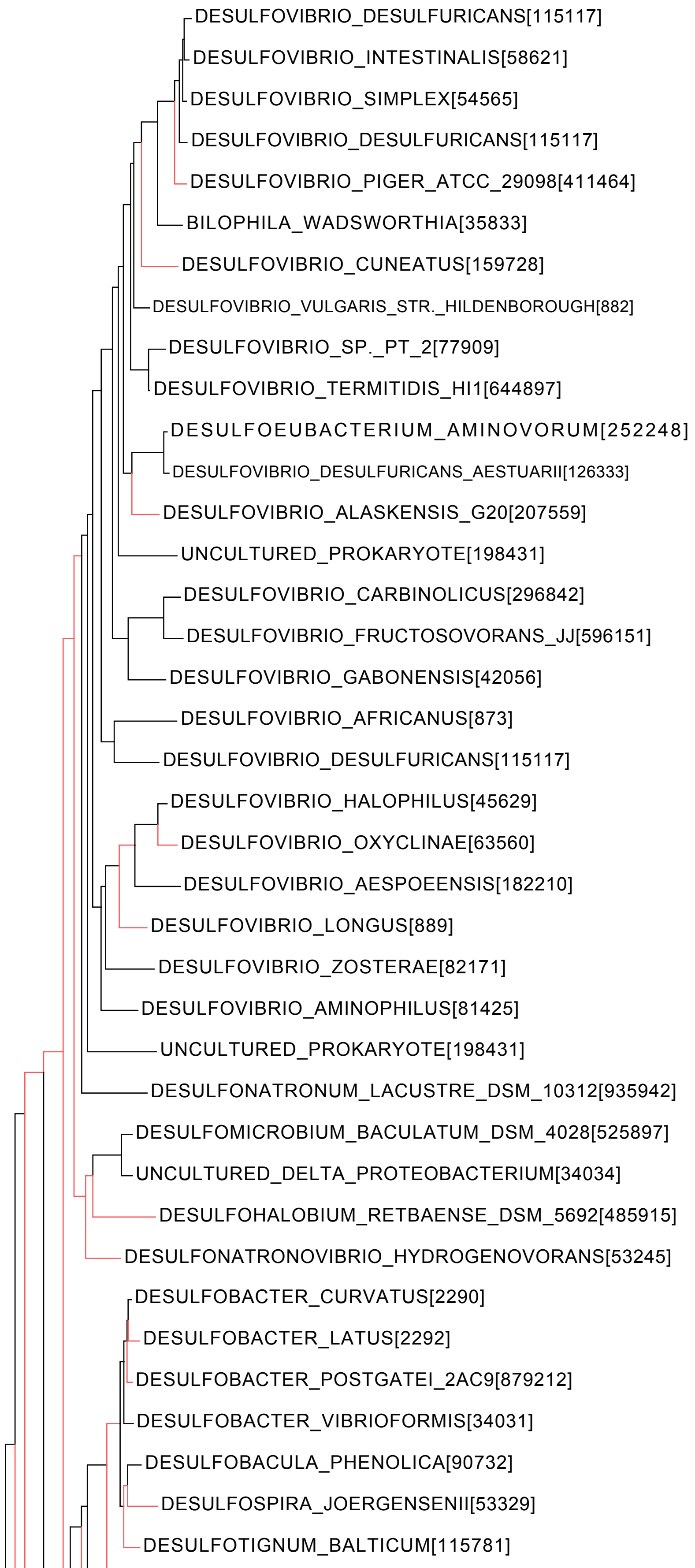

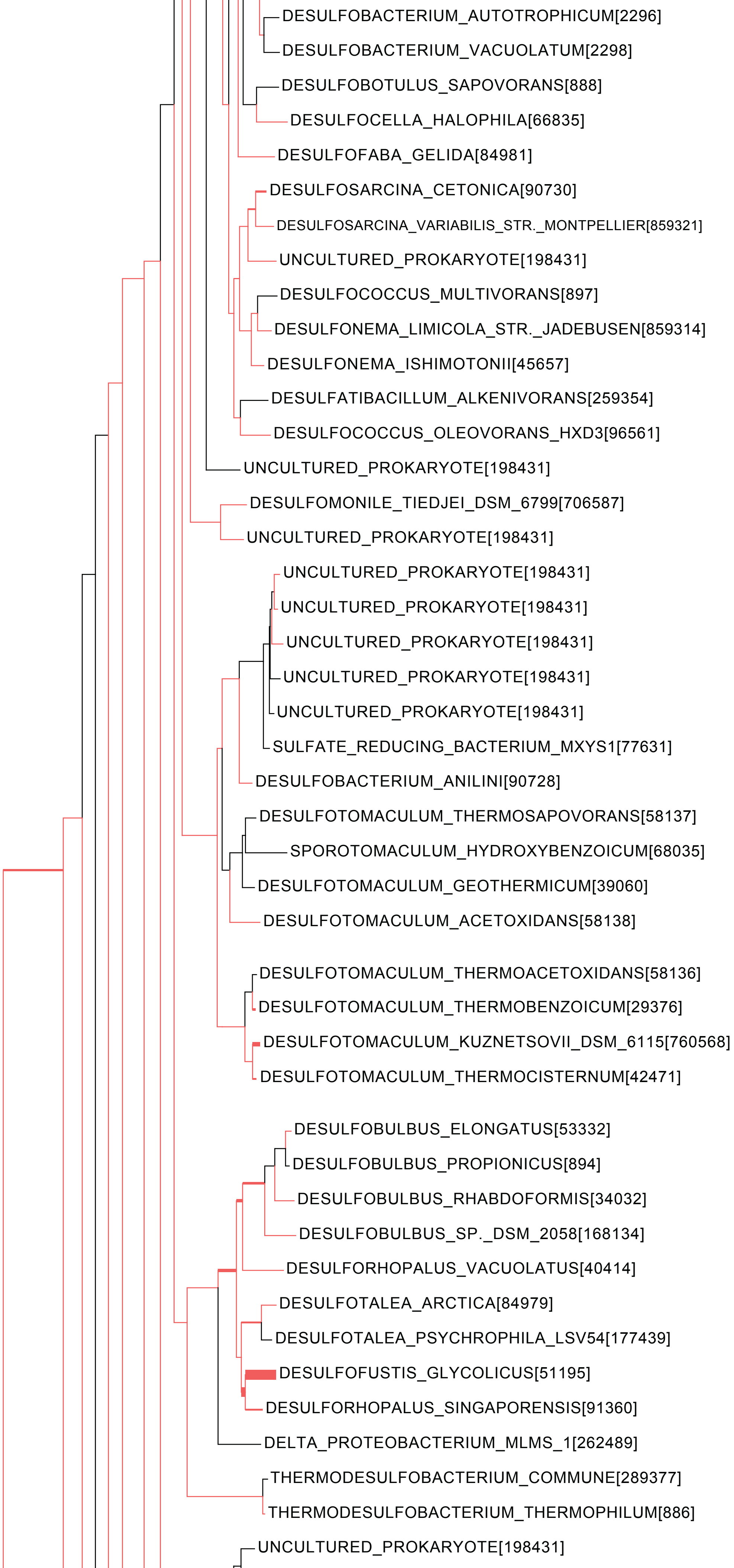
DESULFOBACTERIUM_AUTOTROPHICUM[2296]
DESULFOBACTERIUM_VACUOLATUM[2298]
DESULFOBOTULUS_SAPOVORANS[888]
DESULFOCELLA_HALOPHILA[66835]
DESULFOFABA_GELIDA[84981]
DESULFOSARCINA_CETONICA[90730]
DESULFOSARCINA_VARIABILIS_STR._MONTPELLIER[859321]
UNCULTURED_PROKARYOTE[198431]
DESULFOCOCCUS_MULTIVORANS[897]
DESULFONEMA_LIMICOLA_STR._JADEBUSEN[859314]
DESULFONEMA_ISHIMOTONII[45657]
DESULFATIBACILLUM_ALKENIVORANS[259354]
DESULFOCOCCUS_OLEOVORANS_HXD3[96561]
UNCULTURED_PROKARYOTE[198431]
DESULFOMONILE_TIEDJEI_DSM_6799[706587]
UNCULTURED_PROKARYOTE[198431]
UNCULTURED_PROKARYOTE[198431]
UNCULTURED_PROKARYOTE[198431]
UNCULTURED_PROKARYOTE[198431]
UNCULTURED_PROKARYOTE[198431]
UNCULTURED_PROKARYOTE[198431]
SULFATE_REDUCING_BACTERIUM_MXYS1[77631]
DESULFOBACTERIUM_ANILINI[90728]
DESULFOTOMACULUM_THERMOSAPOVORANS[58137]
SPOROTOMACULUM_HYDROXYBENZOICUM[68035]
DESULFOTOMACULUM_GEOTHERMICUM[39060]
DESULFOTOMACULUM_ACETOXIDANS[58138]
DESULFOTOMACULUM_THERMOACETOXIDANS[58136]
DESULFOTOMACULUM_THERMOBENZOICUM[29376]
DESULFOTOMACULUM_KUZNETSOVII_DSM_6115[760568]
DESULFOTOMACULUM_THERMOCISTERNUM[42471]
DESULFOBULBUS_ELONGATUS[53332]
DESULFOBULBUS_PROPIONICUS[894]
DESULFOBULBUS_RHABDOFORMIS[34032]
DESULFOBULBUS_SP._DSM_2058[168134]
DESULFORHOPALUS_VACUOLATUS[40414]
DESULFOTALEA_ARCTICA[84979]
DESULFOTALEA_PSYCHROPHILA_LSV54[177439]
DESULFOFUSTIS_GLYCOLICUS[51195]
DESULFORHOPALUS_SINGAPORENSIS[91360]
DELTA_PROTEOBACTERIUM_MLMS_1[262489]
THERMODESULFOBACTERIUM_COMMUNE[289377]
THERMODESULFOBACTERIUM_THERMOPHILUM[886]
UNCULTURED_PROKARYOTE[198431]

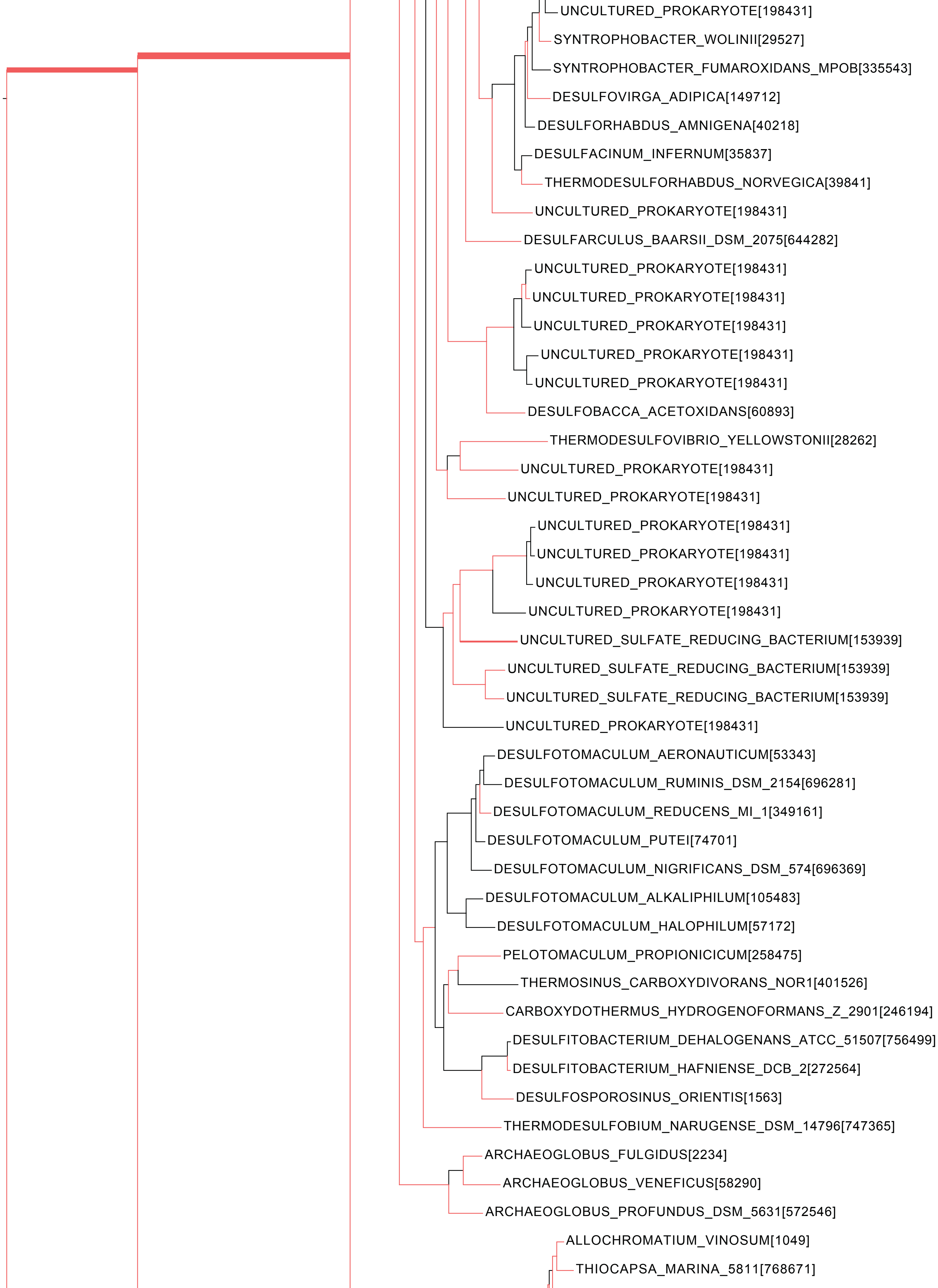
UNCULTURED_PROKARYOTE[198431]
SYNTROPHOBACTER_WOLINII[29527]
SYNTROPHOBACTER_FUMAROXIDANS_MPOB[335543]
DESULFOVIRGA_ADIPICA[149712]
DESULFORHABDUS_AMNIGENA[40218]
DESULFACINUM_INFERNUM[35837]
THERMODESULFORHABDUS_NORVEGICA[39841]
UNCULTURED_PROKARYOTE[198431]
DESULFARCULUS_BAARSII_DSM_2075[644282]
UNCULTURED_PROKARYOTE[198431]
UNCULTURED_PROKARYOTE[198431]
UNCULTURED_PROKARYOTE[198431]
UNCULTURED_PROKARYOTE[198431]
UNCULTURED_PROKARYOTE[198431]
DESULFOBACCA_ACETOXIDANS[60893]
THERMODESULFOVIBRIO_YELLOWSTONII[28262]
UNCULTURED_PROKARYOTE[198431]
UNCULTURED_PROKARYOTE[198431]
UNCULTURED_PROKARYOTE[198431]
UNCULTURED_PROKARYOTE[198431]
UNCULTURED_PROKARYOTE[198431]
UNCULTURED_PROKARYOTE[198431]
UNCULTURED_SULFATE_REDUCING_BACTERIUM[153939]
UNCULTURED_SULFATE_REDUCING_BACTERIUM[153939]
UNCULTURED_SULFATE_REDUCING_BACTERIUM[153939]
UNCULTURED_PROKARYOTE[198431]
DESULFOTOMACULUM_AERONAUTICUM[53343]
DESULFOTOMACULUM_RUMINIS_DSM_2154[696281]
DESULFOTOMACULUM_REDUCENS_MI_1[349161]
DESULFOTOMACULUM_PUTEI[74701]
DESULFOTOMACULUM_NIGRIFICANS_DSM_574[696369]
DESULFOTOMACULUM_ALKALIPHILUM[105483]
DESULFOTOMACULUM_HALOPHILUM[57172]
PELOTOMACULUM_PROPIONICICUM[258475]
THERMOSINUS_CARBOXYDIVORANS_NOR1[401526]
CARBOXYDOTHERMUS_HYDROGENOFORMANS_Z_2901[246194]
DESULFITOBACTERIUM_DEHALOGENANS_ATCC_51507[756499]
DESULFITOBACTERIUM_HAFNIENSE_DCB_2[272564]
DESULFOSPOROSINUS_ORIENTIS[1563]
THERMODESULFOBIUM_NARUGENSE_DSM_14796[747365]
ARCHAEOGLOBUS_FULGIDUS[2234]
ARCHAEOGLOBUS_VENEFICUS[58290]
ARCHAEOGLOBUS_PROFUNDUS_DSM_5631[572546]
ALLOCHROMATIUM_VINOSUM[1049]
THIOCAPSA_MARINA_5811[768671]

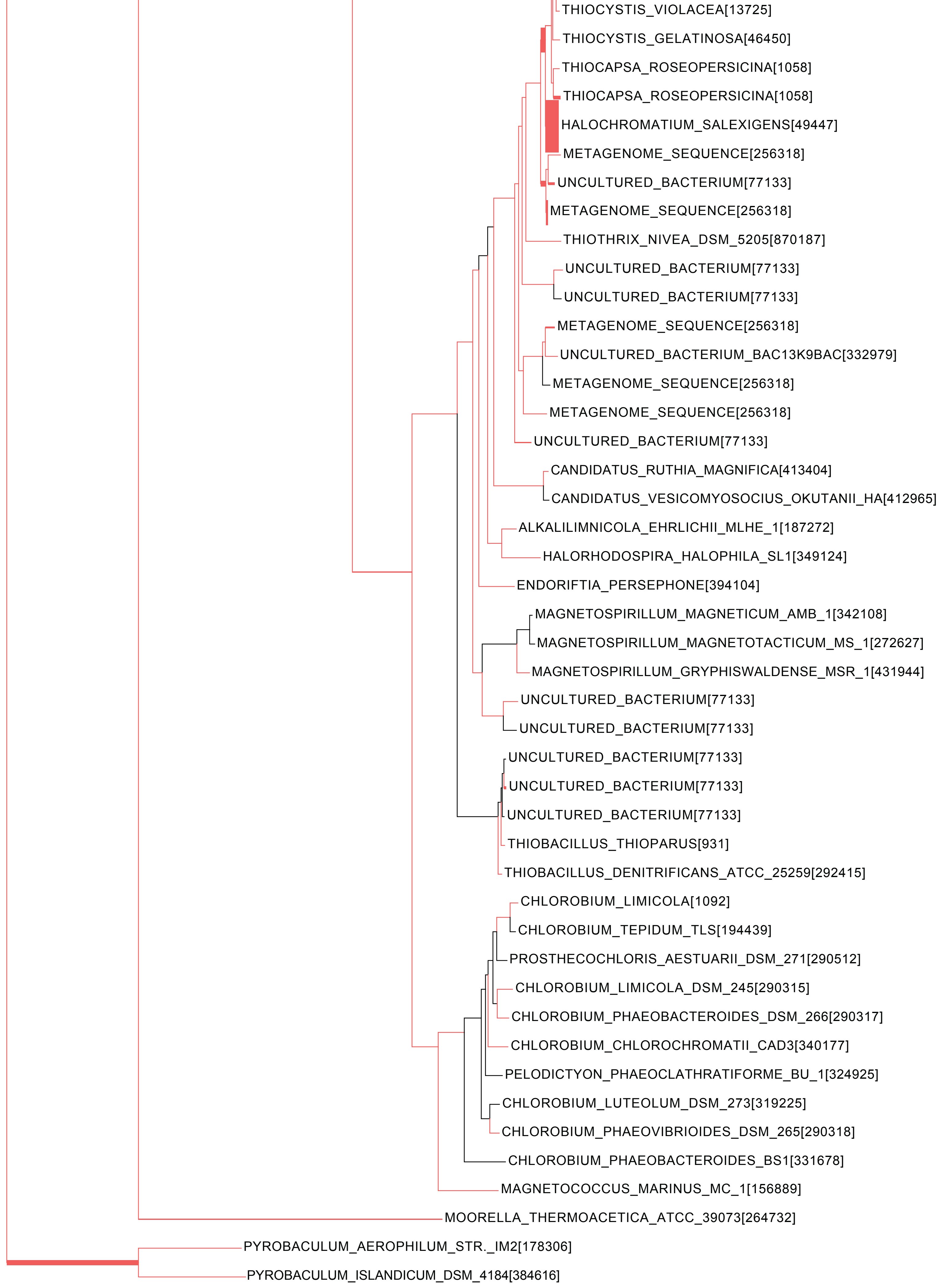
THIOCYSTIS_VIOLACEA[13725]
THIOCYSTIS_GELATINOSA[46450]
THIOCAPSA_ROSEOPERSICINA[1058]
THIOCAPSA_ROSEOPERSICINA[1058]
HALOCHROMATIUM_SALEXIGENS[49447]
METAGENOME_SEQUENCE[256318]
UNCULTURED_BACTERIUM[77133]
METAGENOME_SEQUENCE[256318]
THIOTHRIX_NIVEA_DSM_5205[870187]
UNCULTURED_BACTERIUM[77133]
UNCULTURED_BACTERIUM[77133]
METAGENOME_SEQUENCE[256318]
UNCULTURED_BACTERIUM_BAC13K9BAC[332979]
METAGENOME_SEQUENCE[256318]
METAGENOME_SEQUENCE[256318]
UNCULTURED_BACTERIUM[77133]
CANDIDATUS_RUTHIA_MAGNIFICA[413404]
CANDIDATUS_VESICOMYOSOCIUS_OKUTANII_HA[412965]
ALKALILIMNICOLA_EHRLICHII_MLHE_1[187272]
HALORHODOSPIRA_HALOPHILA_SL1[349124]
ENDORIFTIA_PERSEPHONE[394104]
MAGNETOSPIRILLUM_MAGNETICUM_AMB_1[342108]
MAGNETOSPIRILLUM_MAGNETOTACTICUM_MS_1[272627]
MAGNETOSPIRILLUM_GRYPHISWALDENSE_MSR_1[431944]
UNCULTURED_BACTERIUM[77133]
UNCULTURED_BACTERIUM[77133]
UNCULTURED_BACTERIUM[77133]
UNCULTURED_BACTERIUM[77133]
UNCULTURED_BACTERIUM[77133]
THIOBACILLUS_THIOPARUS[931]
THIOBACILLUS_DENITRIFICANS_ATCC_25259[292415]
CHLOROBIUM_LIMICOLA[1092]
CHLOROBIUM_TEPIDUM_TLS[194439]
PROSTHECOCHLORIS_AESTUARII_DSM_271[290512]
CHLOROBIUM_LIMICOLA_DSM_245[290315]
CHLOROBIUM_PHAEOBACTEROIDES_DSM_266[290317]
CHLOROBIUM_CHLOROCHROMATII_CAD3[340177]
PELODICTYON_PHAEOCLATHRATIFORME_BU_1[324925]
CHLOROBIUM_LUTEOLUM_DSM_273[319225]
CHLOROBIUM_PHAEOVIBRIOIDES_DSM_265[290318]
CHLOROBIUM_PHAEOBACTEROIDES_BS1[331678]
MAGNETOCOCCUS_MARINUS_MC_1[156889]
MOORELLA_THERMOACETICA_ATCC_39073[264732]
PYROBACULUM_AEROPHILUM_STR._IM2[178306]
PYROBACULUM_ISLANDICUM_DSM_4184[384616]

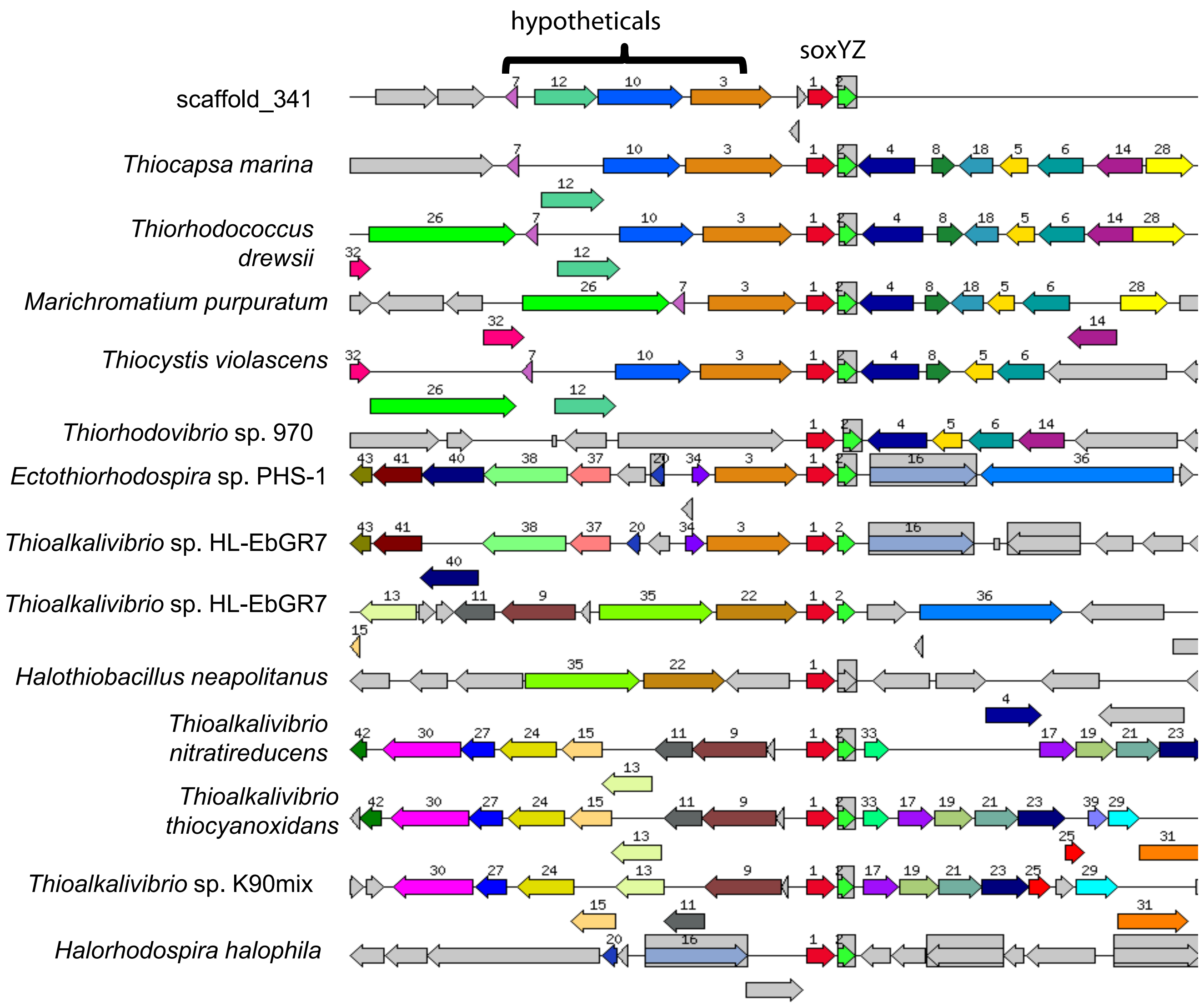


Supplemental Figure 6. *Chromatiales soxYZ* gene. Generated by RAST synteny viewer using 1e-20 BLASTP similarity cutoff to define and color gene sets (each set has unique number and color). Ordered according to % aa similarity to scaffold_341 soxY query. Note the conserved *Chromatiales* hypothetical gene cluster upstream from *soxYZ* (7,12,10,3).

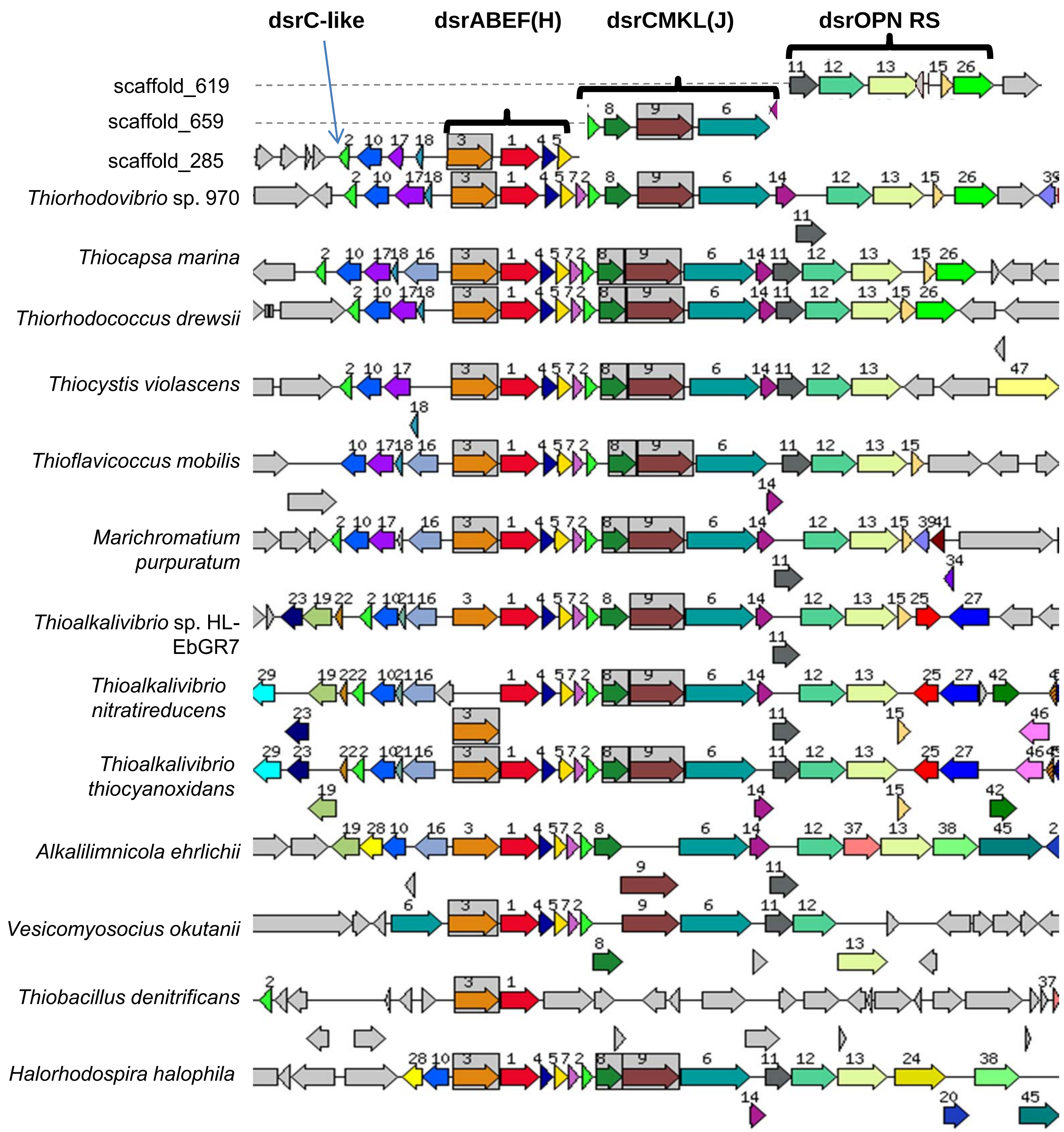


**Supplemental Figure 7.** *Chromatiales dsrABEFHCMKLJOPNRS* gene cluster is represented in three distinct metagenomic contigs from PB-PSB1. Generated by RAST synteny viewer using 1e-20 BLASTP similarity cutoff to define and color gene sets (each set has unique number and color). Ordered according to % aa similarity to scaffold_285 *dsrA* query. Genes shown in parenthesis at top (*dsrH* and *dsrJ*) were not annotated on metagenomic contigs by RAST but are identifiable as gene-fragments using independent BLASTP queries. Also, note the conserved *Chromatiales* hypothetical gene cluster upstream from *dsrAB* (10,17,16/18).

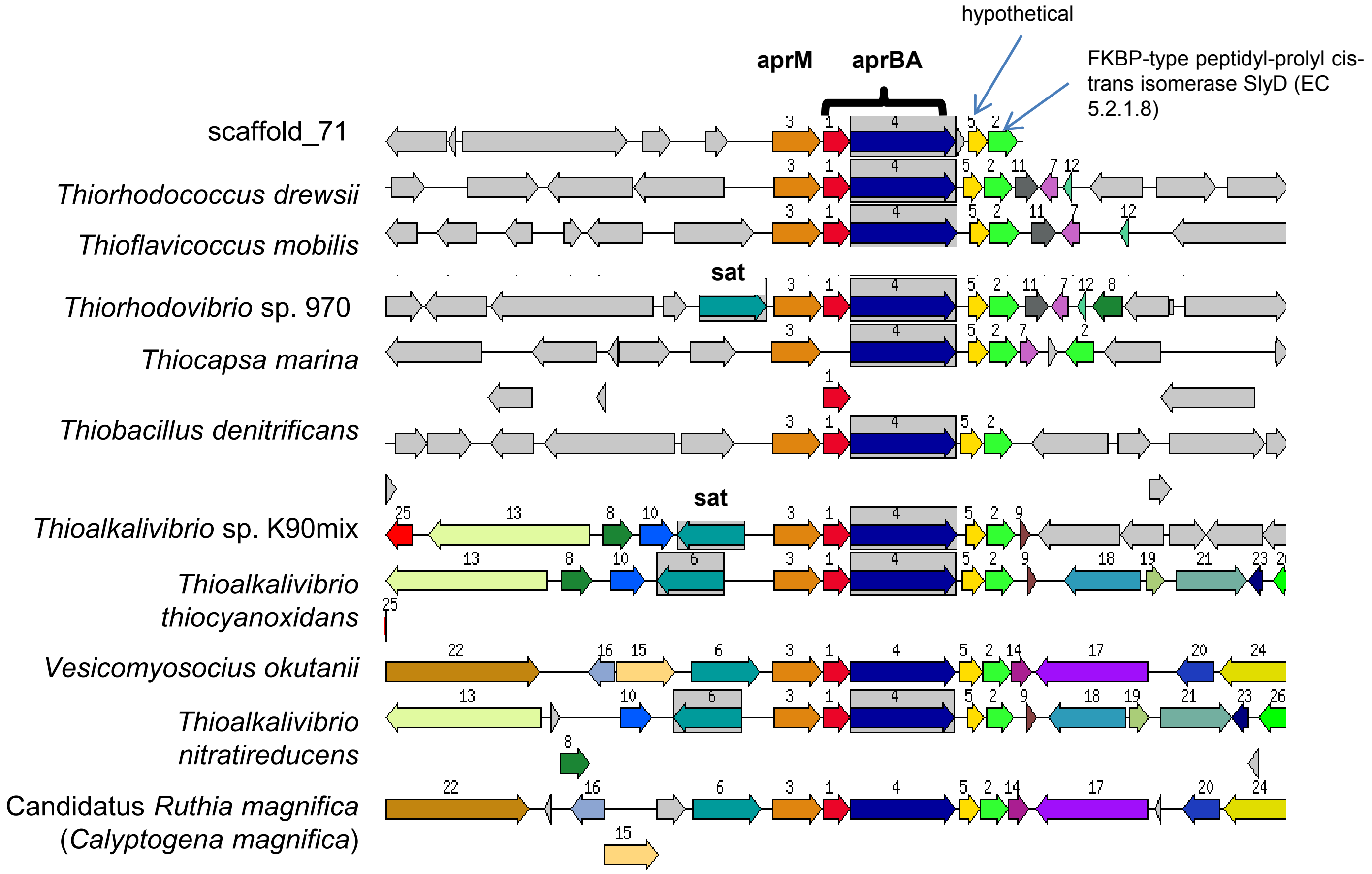


**Supplemental Figure 8.** Genomic context of *aprM-aprBA* gene cluster in PB-PSB1. Generated by RAST synteny viewer using 1e-20 BLASTP similarity cutoff to define and color gene sets (each set has unique number and color). Ordered according to % aa similarity to the scaffold_71 *aprB* query.

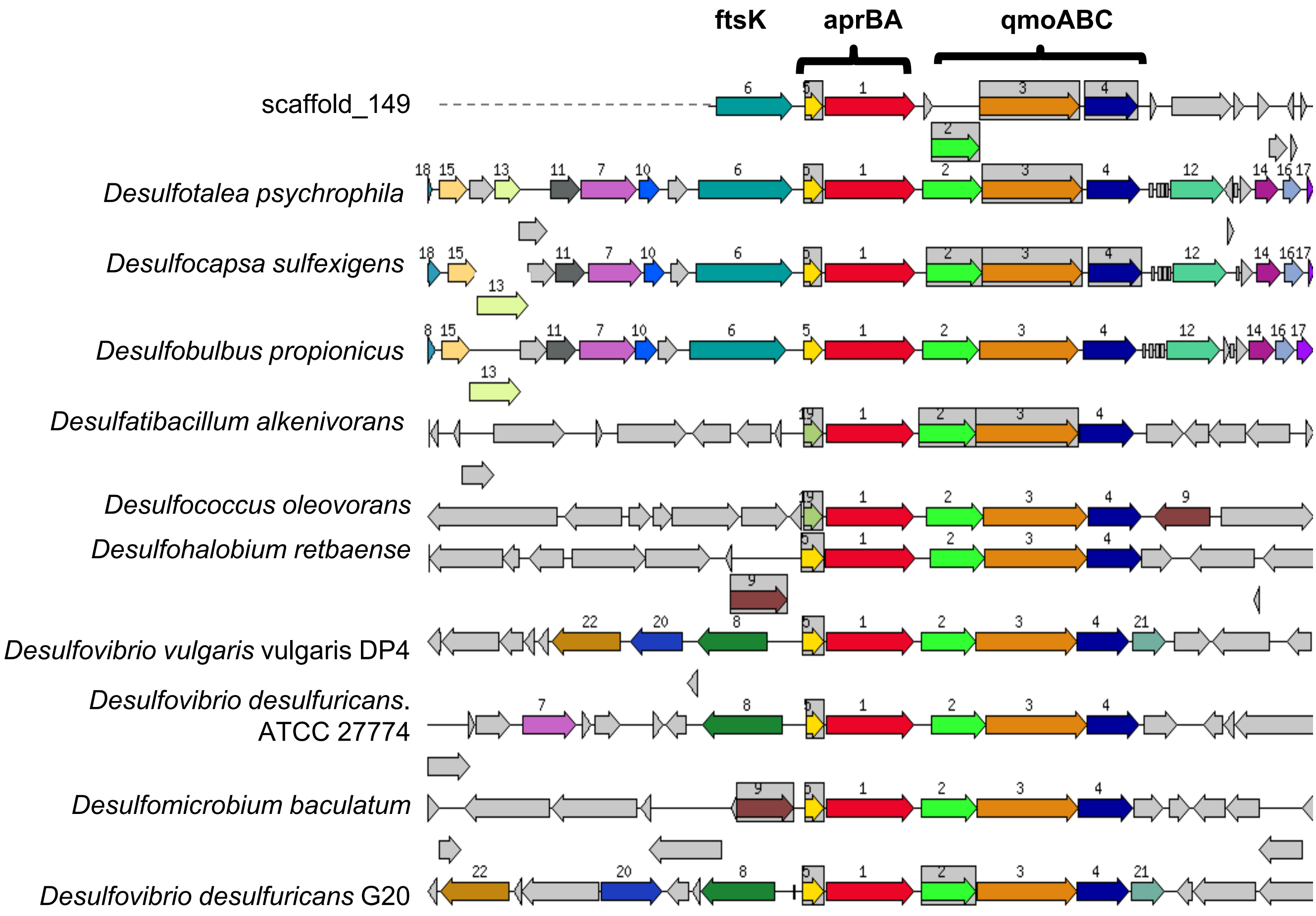


**Supplemental Figure 9.** Genomic context of *aprAB-qmoABC* gene cluster in PB-SRB1. Generated by RAST synteny viewer using 1e-20 BLASTP similarity cutoff to define and color gene sets (each set has unique number and color). Ordered according to % aa similarity to the scaffold 149 *aprA* query.

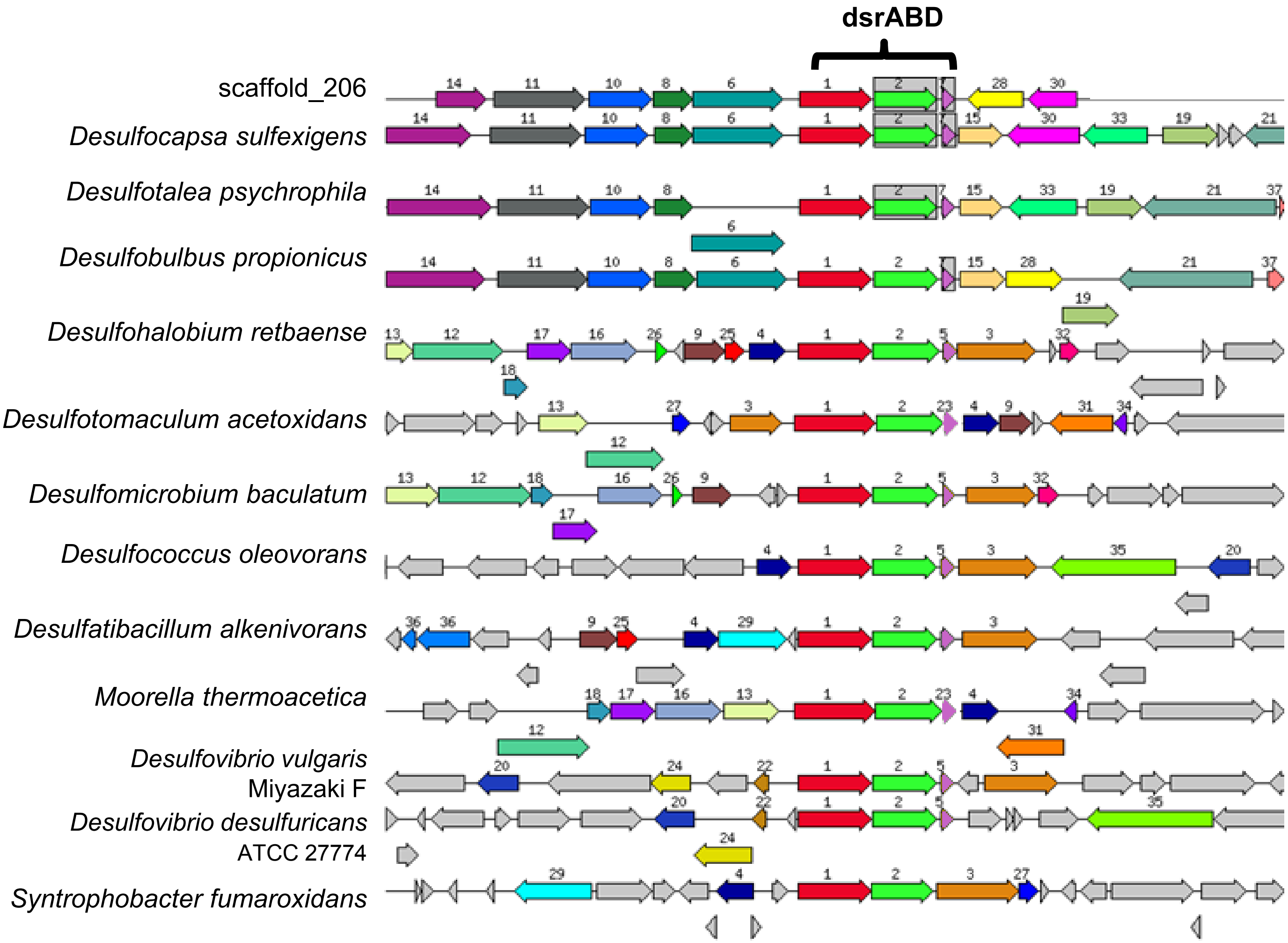


**Supplemental Figure 10.** Genomic context of *dsrAB* PB-SRB1. Generated by RAST synteny viewer using 1e-20 BLASTP similarity cutoff to define gene sets (each set has unique number and color). Table below shows annotations for *Desulfobulbaceae*-conserved genes in this genomic region. Ordered according to % aa similarity to scaffold 206 *dsrA* query.

| Set | Gene annotation |
|---|---|
| 6 | Glucose-6-phosphate isomerase (EC 5.3.1.9) |
| 8 | Ribulose-phosphate 3-epimerase (EC 5.1.3.1) |
| 10 | Rossmann fold nucleotide-binding protein Smf |
| 11 | RNA polymerase sigma factor RpoD |
| 14 | DNA primase (EC 2.7.7.-) |
| 15 | Biotin-protein ligase (EC 6.3.4.15) |
| 28 | Alcohol dehydrogenase (EC 1.1.1.1) |
| 30 | S-adenosylmethionine synthetase (EC 2.5.1.6 |

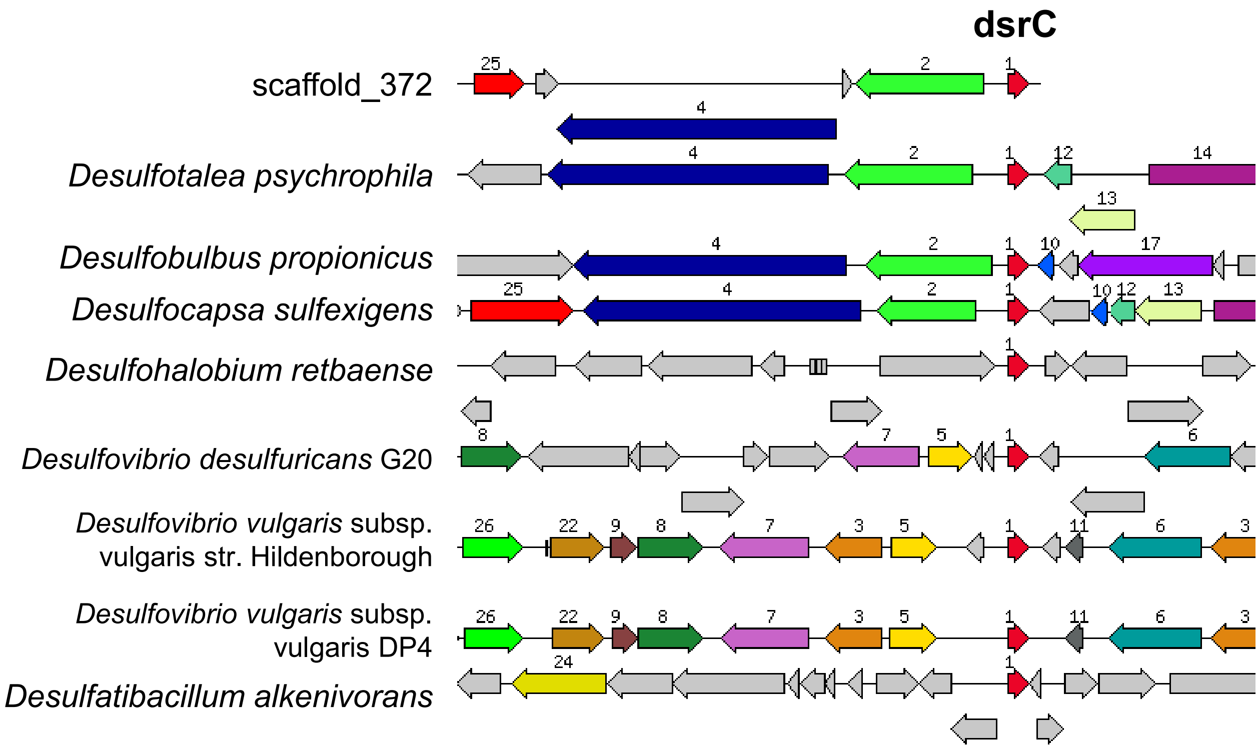


**Supplemental Figure 11.** Genomic context of *dsrC* PB-SRB1. Generated by RAST synteny viewer using 1e-20 BLASTP similarity cutoff to define gene sets (each set has unique number and color). Table below shows annotations for conserved genes in PB-SRB1 in this genomic region. Ordered according to % aa similarity to the scaffold_372 *dsrC* query.

| Set | Annotated function |
|---|---|
| 1 | dsrC |
| 2 | Peptidyl-prolyl cis-trans isomerase ppiD (EC 5.2.1.8) |
| 4 | COG0553: Superfamily II DNA/RNA helicases, SNF2 family |
| 25 | Uroporphyrinogen-III methyltransferase (EC 2.1.1.107) / Uroporphyrinogen-III synthase (EC 4.2.1.75) |

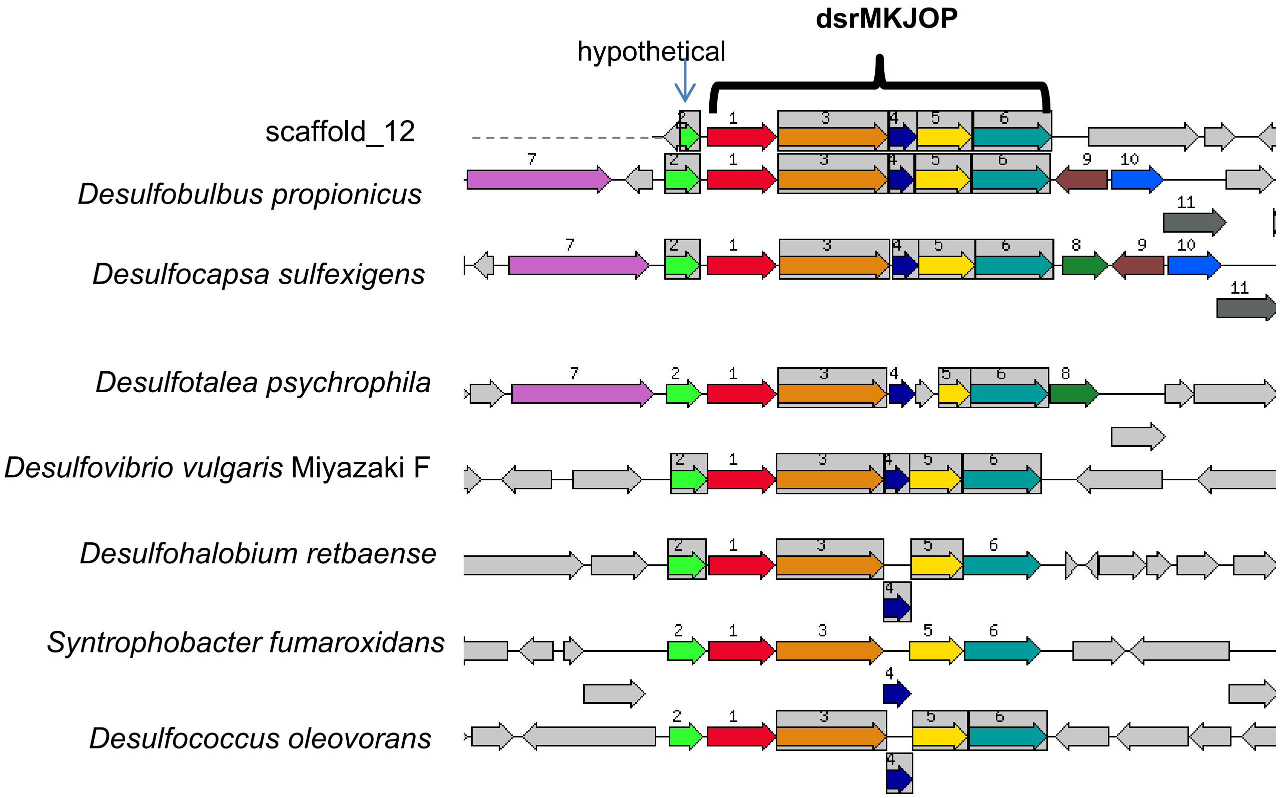


**Supplemental Figure 12.** Genomic context of *dsrMKJOP* gene cluster in PB-SRB1. Generated by RAST synteny viewer using 1e-20 BLASTP similarity cutoff to define and color gene sets (each set has unique number and color). . Ordered according to % aa similarity to the scaffold_12 *dsrM* query.

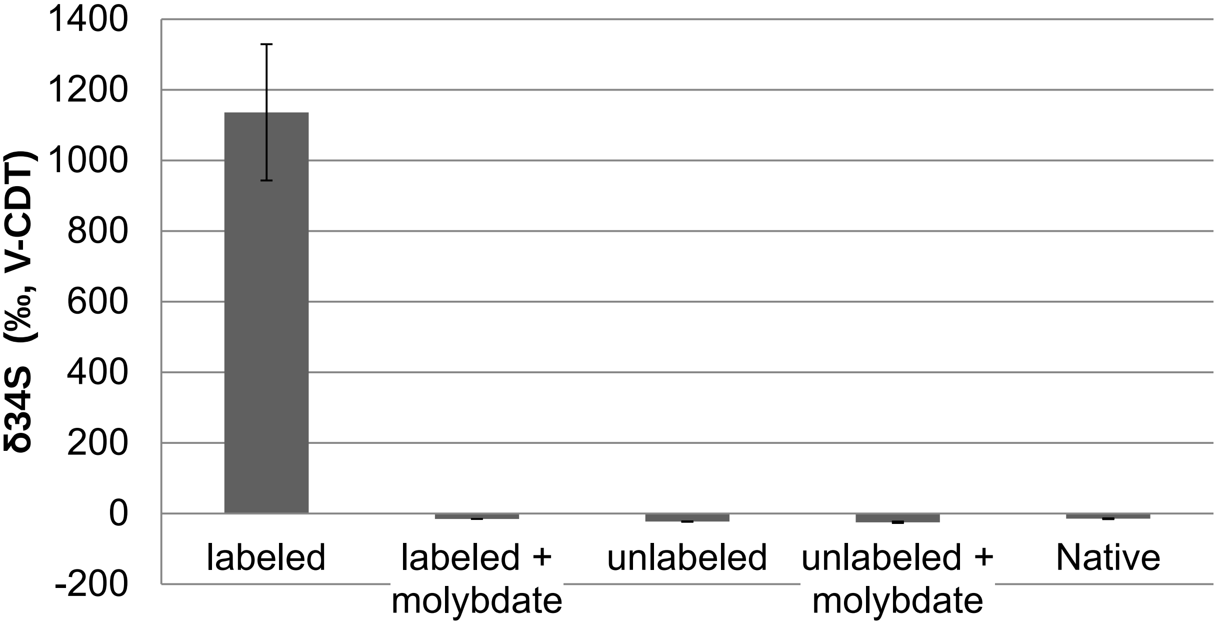


| ‰ V-CDT | labeled | labeled + molybdate | unlabeled | unlabeled + molybdate | Native |
|---|---|---|---|---|---|
| **mean** | 1135.98 | -15.42 | -22.88 | -24.86 | -14.8 |
| **standard deviation** | 193 | 0.00 | 0.27 | 2.03 | 1.19 |
| **Biological replicates** | 3 | 2 | 3 | 2 | 3 |

**Supplemental Figure 13**. Elemental analyzer-isotope ratio mass spectrometry (EA-IRMS) of pooled berry biomass from dark incubations with either added $^{34}$S-enriched sulfate ("labelled") or standard sulfate ("unlabeled"). Incubations were conducted both with and without 10 mM molybdate. Also shown are three different measurements of pooled berry biomass taken directly from the marsh ("native"). Values shown are per mil V-CDT the mean of biological replicates, error bars show standard deviation between these replicates.
